# Biomechanics of Osseointegration of a Dental Implant in the Mandible Under Shock Wave Therapy: In Silico Study

**DOI:** 10.3390/ma17246204

**Published:** 2024-12-19

**Authors:** Alexey Smolin, Galina Eremina, Irina Martyshina, Jing Xie

**Affiliations:** 1 Institute of Strength Physics and Materials Science, Siberian Branch of the Russian Academy of Sciences, Pr. Akademicheskii, 2/4, 634055 Tomsk, Russia; anikeeva@ispms.ru (G.E.); mira@ispms.ru (I.M.); 2 State Key Laboratory of Explosion Science and Technology, Beijing Institute of Technology, Beijing 100081, China; jxie@bit.edu.cn

**Keywords:** extracorporeal shock wave therapy, dental implant osseointegration, poroelasticity, discrete element modeling

## Abstract

The most time-consuming aspect of dental prosthesis installation is the osseointegration of a metal implant with bone tissue. The acceleration of this process may be achieved through the use of extracorporeal shock wave therapy. The objective of this study is to investigate the conditions for osseointegration of the second premolar implant in the mandibular segment through the use of a poroelastic model implemented in the movable cellular automaton method. The mandibular segment under consideration includes a spongy tissue layer, 600 µm in thickness, covered with a cortical layer, 400 µm in thickness, and a gum layer, 400 µm in thickness. Furthermore, the periodontal layers of the roots of the first premolar and first molar were considered, while the implant of the second premolar was situated within a shell of specific tissue that corresponded to the phase of osseointegration. The model was subjected to both physiological loading and shock wave loading across the three main phases of osseointegration. The resulting fields of hydrostatic pressure and interstitial fluid pressure were then subjected to analysis in accordance with the mechanobiological principles. The results obtained have indicated that low-intensity shock wave therapy can accelerate and promote direct osseointegration: 0.05–0.15 mJ/mm^2^ in the first and second phases and less than 0.05 mJ/mm^2^ in the third phase. In comparison to physiological loads (when bone tissue regeneration conditions are observed only around the implant distal end), shock waves offer the primary advantage of creating conditions conducive to osseointegration along the entire surface of the implant simultaneously. This can significantly influence the rate of implant integration during the initial osteoinduction phase and, most crucially, during the longest final phase of bone remodeling.

## 1. Introduction

The longest and most significant phase in the installation of a dental prosthesis is osseointegration of its metal implant into bone tissue with the establishment of a functional connection between them. Therefore, it is important to speed up this process [1].

It has been established that the osseointegration of a dental implant occurs according to the scenario of differentiation of osteoprogenitor cells (osteogenic cells, mesenchymal stem cells) into osteoblasts with subsequent intramembranous ossification and without formation of cartilage tissue [2]. Another type of ossification, endochondral ossification, is typical of fracture healing and occurs inside cartilage germs when progenitor cells differentiate into chondroblasts [3]. Many researchers believe that the main processes of dental implant osseointegration in the jaw tissues take place at the scale of hormones, proteins, and cells (micro-scale) [4]. Moreover, the surface properties of the implant and new methods of surface treatment additionally stimulate the osteogenic cell response [5] that leads to specific history of new bone density and elastic properties [6]. Another possible mechanism for stimulating cell response is the use of electrical signals, which can be generated by piezoelectric scaffolds [7] that are specially designed and manufactured using additive technologies [8].

Depending on the final tissue–implant interface, three types of implant integration can be realized: osseointegration (direct connection between the implant and the bone tissue), fibro-osseous integration (involving a fibrous layer between the bone and the implant), and connective tissue integration (when the implant surface is surrounded by fibrous connective tissue) [9]. Osseointegration is the ideal case, but often fibro-osseous integration occurs in at least part of the entire interface. The last case (connective tissue integration) is considered a negative scenario in dentistry and is particularly common in orthopedic surgery.

In the literature, the following three phases of implant integration are distinguished [10]: (i) osteoconduction (hemostatic phase with blood clot formation), which lasts up to several weeks; (ii) osteoinduction (phase of new/immature woven bone formation), which lasts 1–2 months; (iii) a long-term remodeling phase characterized by the restructuring of woven bone into mature bone tissue, which lasts several months.

In the first phase of implant integration, the space around the implant is filled by a blood clot with a meshwork of fibrin fibers (primary matrix) [11]. Fibrin fibers provide the opportunity for osteogenic cells to migrate to the surface of the implant under the influence of growth factors. Growth factors (signals) attract fibroblasts and other undifferentiated cells to the zone of fibrin matrix and stimulate their differentiation. This stage is the most important (crucial) because it lays the foundation for the formation of the future type of implant integration (the peculiarities of its course largely determine the further bone–implant interface). Tight adhesion of the blood clot to the surface of the implant and formation of fibrin “bridges” of 0.1–0.2 mm between it and the bone create conditions for proliferation of osteogenic cells along the fibrin fibers to the implant and formation of bone on the surface of the implant itself. Most likely, the processes of direct and distance (indirect) osteogenesis occur in parallel at different parts of the bone–implant interface.

In the second phase of implant integration, the surrounding area consists of a disordered mineralized tissue, also called granulation tissue or osteoid, i.e., highly vascularized tissue made of fibroblasts, osteoblasts, and an extracellular matrix. The mineralization of the osteoid by the hormonal stimulation of local calcium and phosphate ions occurs to form woven bone.

The remodeling phase, which ends up with the formation of mature tissue, begins with the resorption of woven bone. It should be noted that the elasticity parameters of new bone are lower than those of mature bone tissue, and the permeability is higher. During this period, mechanical stress is highly important for the formation of bone tissue capable of withstanding it. Therefore, it is crucial to know how stress and strain are distributed around the implant and change under physiological loading and progressing osseointegration.

Due to activation role of mechanical stress in cellular signaling pathways, it is also possible to use additional mechanical stimuli to control the implant osseointegration. Thus, in [12], it was proposed that shock wave treatment should be used to improve dental implant osseointegration. In [13], the author concluded that this treatment’s benefits (anti-inflammation, bone regeneration, microbial disruption) could be a favorable therapeutic modality for peri-implantitis. To date, in dentistry, the use of extracorporeal shock wave therapy (ESWT) has been proposed for the treatment of caries and periodontal disease [14], which was confirmed in a humanized rat model [15], and for the regeneration of alveolar bone tissue in general degenerative diseases [16], which was later reviewed in detail in [17]. Since 2010, the possibility of using shock wave therapy in the pre- and post-implantation periods of dental surgery has been proposed for its antibacterial effect [18]. This effect was later actively investigated in [19]. To the best of our knowledge, the effects of ESWT on the anabolic response of biological tissues to a dental implant, including its anti-inflammatory effect, angiogenesis, and bone regeneration in the area in close contact with a dental implant, have not been studied.

In [20], the authors compared two types (collimated and focused) of low-intensity pulsed ultrasound stimulation on the mandible implants in rabbits. Bone ingrowth in different directions was observed. The bone mass and mean pore occupancy fraction in the “focused” group were much higher than in the “collimated” group. The new bone formation in the different sides of the implants in the “focused” group was greater than in the “collimated” and control groups.

Most in vivo and in vitro studies of implant osseointegration have been performed on small and medium animals: rats, rabbits, and dogs [21]. The main drawback of such studies is the lack of long-term observation. In addition, studies conducted on small animals with a short lifespan do not allow us to take into account pathological conditions similar to those in humans (osteoporosis of the jaw, etc.). In silico predictions of bone tissue regeneration under the influence of mechanical stimuli are mainly performed within the framework of computational methods of continuum mechanics (finite element analysis) using an elastic model for bone tissue [22]. However, the bone tissue, as well as the other tissues surrounding the implant, are a fluid saturated material and exhibit a viscoelastic mechanical behavior. In [23], a numerical coupled poroelastic and biochemical model was developed with a stochastic analysis of the bone tissue in the area of its contact with the dental implant. The conditions for bone regeneration were analyzed using bone density. The analysis of bone tissue regeneration around the implant based on poroelasticity and a mechanoregulatory model was presented in [24]. In [25], this model was used to predict healing pattern around implants of different geometry of implant thread design. The prediction of conditions for bone tissue regeneration around the implant based on a poroelastic mechanoregulatory model is also presented in [26]. In [27], the authors proposed a poroviscoelastic mechanoregulatory model and studied the interaction of the implant with the biomaterial and the functional adaptation of the bone tissue under a load that varied slowly over time (masticatory load), which produces a non-local mechanical stimulus consisting of a linear combination of deformation energy and viscous dissipation. The above studies, based on mechanoregulatory models, are attractive because they predict the history of changes in bone density and elastic modulus. The disadvantage of these models for the purpose of this study, in our opinion, is that the coefficients of their phenomenological equations are experimentally determined only for slow physiological loading of certain intensity and mainly for endochondral ossification during fracture healing of axial bones. These coefficients, and perhaps even the equations themselves (due to differences in ossification of the skull and limbs [3]), are not valid for other types of mechanical loading, especially for ESWT exposure of the mandible.

To the best of our knowledge, the modeling of osseointegration of a dental implant under the conditions of low-energy acoustic action based on a poroelastic model, taking into account human physiological characteristics, has not yet been carried out. Therefore, the aim of this work is to numerically study the influence of ESWT on the osseointegration processes of an implant in the human mandible based on a poroelastic model.

As a modeling method, the movable cellular automaton (MCA) method was adopted, which is representative of computational particle mechanics and makes it possible to correctly describe the mechanical behavior of poroelastic heterogeneous materials. First of all, the developed 3D numerical model of the mandibular segment with an implant was verified and validated. After that, numerical studies of the stress state of the model under physiological loading and shock wave exposure were carried out. Finally, we discussed the results obtained here and compared them with available data from the literature.

## 2. Materials and Methods

**Figure 1:**
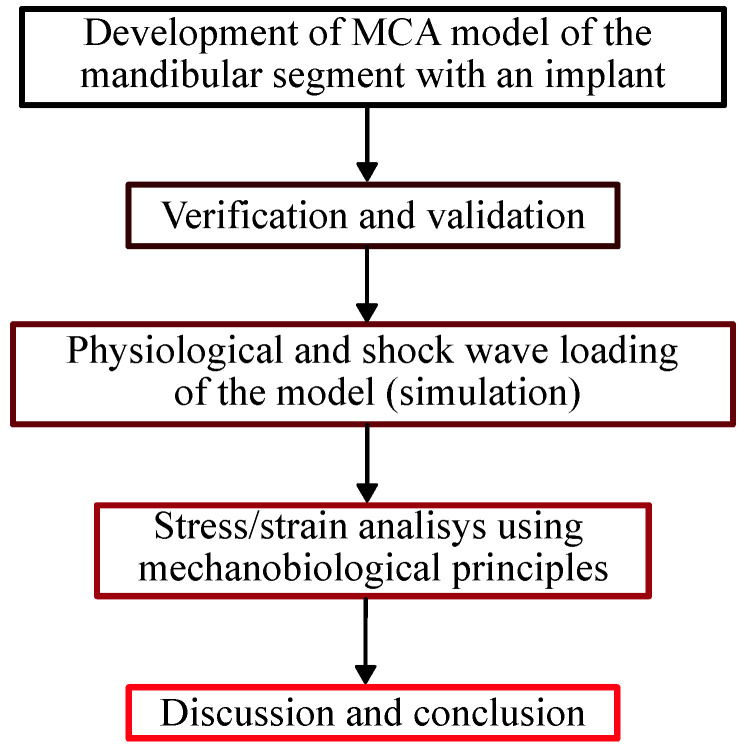
Flowchart of the study.

The flowchart of the study is depicted in Figure 1. First, the developed model of the mandibular segment was verified and validated. Verification was performed by analyzing the convergence of the model stiffness as the size of the automaton decreases. Validation was performed by comparing the model stiffness with experimental data. Then, we simulated the mechanical behavior of the model under physiological loading (mastication) and shock wave exposure. The simulation results were analyzed on the basis of mechanobiological principles, which allowed us to draw some conclusions about what type of tissue is formed in the peri-implant gap and whether the conditions for normal remodeling of bone tissue around the implant are met. Finally, after discussing the results obtained, we came to the conclusions of the study.

The movable cellular automaton method was used to analyze the mechanical behavior of the human mandibular segment with an implant under extracorporeal shock wave therapeutic treatment. The mandibular segment includes a section from the first premolar to the first molar, with an implant of the second premolar. The simulation results were analyzed based on mechanobiological principles and available information on the effect of external mechanical loading on the biological tissues.

### 2.1. Method of Simulation

We utilized the movable cellular automaton method (MCA), which is an improved version of the discrete element method as presented in [28,29,30]. The MCA method models materials as a collection of discrete elements, called movable cellular automata, that interact according to specific rules of multi-body interaction and can change their state. This allows the mechanical behavior of such a system to be treated as deformation of an isotropic elastoplastic body within a particle approach. The motion of the system is described by the Newton–Euler equations for the translation and rotation of each discrete element. The influence of the interstitial fluid contained in the pore space of a discrete element on its stress state is described based on Biot’s linear poroelasticity model. This model adapted to the method of movable cellular automata, has been well established for modeling the mechanical behavior of biomaterials under dynamic loading at the micro-, meso- and macro-scales [30,31,32,33]. Therefore, it was this approach that was used herein to model the mechanical behavior of the biological tissues in the mandibular segment with an implant. Furthermore, the MCA3D version 3.0 software allows the simulation of objects with complex shapes while maintaining precise geometric parameters. The detailed description of the method of movable cellular automata is presented in the Supplementary Materials.

The method was implemented in the in-house code MCA3D, which is written in C++ programming language and utilizes the Qt library for creating models, visualization, and analyzing the simulation results. The MCA3D code has been used in many studies by the authors and their colleagues, the results of which have been published in many papers. In particular, the verification and validation of poroelastic models of the bone tissues based on the MCA method were carried out in [31,32,33].

### 2.2. Model of Mandibular Segment

**Figure 2:**
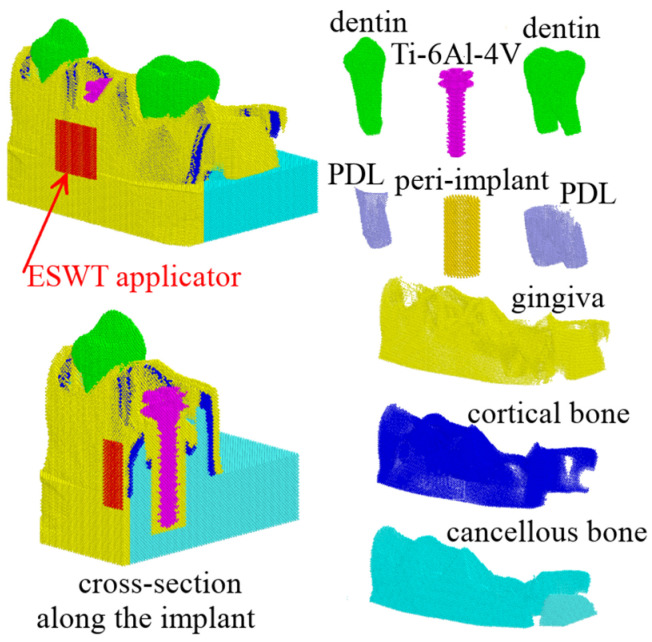
Model of the mandibular segment with an implant of the second premolar and its different parts with corresponding materials.

The mandibular segment model is shown in Figure 2. It included the following: spongiosa (cancellous tissue) covered with a layer of cortical tissue 600 microns thick and a gingiva 400 microns thick; first premolar and second molar with periodontal ligaments (PDL); implant of the second premolar in a cylindrical shell with a radius of 2.5 mm (that corresponds to a 0.5 mm thick peri-implant gap), whose material had different properties at different phases of osseointegration ((i) primary matrix; (ii) granulation tissue and thin fibrous interface with implant; (iii) woven bone).

The geometric solid models of the implant and the mandible parts were taken from the internet. Cutting of the molar section from the mandible solid model, the creation of the lower part of the model, and the shell around the implant was carried out using the open-source Free CAD software version 0.18.

It is well known that biological tissues exhibit anisotropy in their elastic properties. According to Chung and Dechow [34], the difference in elastic moduli in different directions can reach 1.5 times for the cortical bone in the mandible. However, for the purposes of this study, it is much more important to consider the geometry of cortical bone, cancellous bone, and gingiva, because the differences in the properties of these tissues are much higher than those due to anisotropy. Obviously, to enhance the accuracy of the presented model, it would be better to incorporate the anisotropy of elastic properties as well as the permeability of bone tissues.

Biological tissues also demonstrate strain rate dependence in their mechanical behavior, which is typically described as a viscoelastic body. However, it is our understanding that this phenomenon can be more accurately represented by a poroelastic model, which accounts for the pressure of the interstitial fluid, which plays a role in mechanobiological principles.

The poroelastic properties of the tissues used in this study are presented in Table 1 and correspond to the data from the literature [26,35,36,37]. The fluid in biological tissues is assumed to be equivalent to salt water, with a bulk modulus of *K*_f_ = 2.4 GPa, a density of *ρ*_f_ = 1000 kg/m^3^, and a viscosity of *η*_f_ = 1 mPa × s [38]. The properties of the metal parts of the model (implant and shock wave applicator) are presented in Table 2.

**Figure 3:**
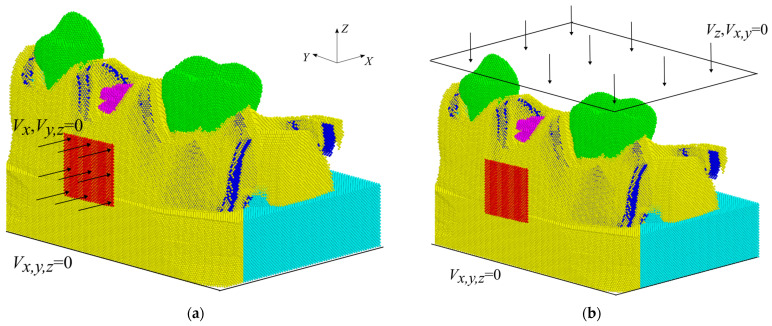
Scheme of loading the segment of the mandible in modeling: (**a**) therapeutic shock wave loading; (**b**) physiological loading.

To simulate shock wave exposure to the mandible segment, a special copper applicator was introduced into the model. The applicator had the shape of a square plate with dimensions of 7 × 7 mm and a thickness of 0.3 mm. It was positioned in the vicinity of the implant as shown in Figure 1 and Figure 3. Shock wave exposure was simulated by moving this applicator along the *X*-axis with the velocity increased smoothly during 0.01 µs up to the maximum value *V* (Figure 3a), which was determined by the energy flux density (EFD) of the shock wave (a detail description of such determination can be found in [32]). The lower layer of the automata of the model was fixed throughout the simulation. The effects of shock waves with energy flux densities ranging from 0.01 to 0.5 mJ/mm^2^ were studied. In total, the load consisted of 5 pulses with an interval of 1 µs each.

To verify, validate, and determine the effectiveness of shock wave exposure on the conditions of implant osseointegration, a numerical study of the physiological loading was also carried out. To achieve this, the lower layer of the automata of the model was fixed, and the upper automata of the model (or rather, the teeth) moved downwards at the same velocity of 0.1 m/s until the resistance to the applied load reached 100 N or 200 N, which corresponds to the conditions at rest and mastication, respectively (Figure 3b).

The therapeutic effect of mechanical action is based on mechanobiological principles that are formulated on the experimental fact that a certain level of mechanical stress and deformation leads to the differentiation a certain type of cells and growth of the corresponding biological tissue [39,40,41,42,43,44,45,46]. The process of osteoblast differentiation (regeneration of bone tissue by intramembranous ossification) is promoted by stresses below 0.15 MPa and a shear strain value of up to 5%. Fibroblast differentiation (regeneration of fibrous tissue) is promoted by tensile stresses higher 0.15 MPa (0.7 MPa is the most favorable value). Chondrocyte differentiation (cartilaginous tissue regeneration and endochondral ossification) is promoted by compressive stresses in the range from 0.15 to 2 MPa and strains higher than 5%. At stresses below 3 kPa, chondrogenesis and osteogenesis do not occur, and compressive stresses of the order of 0.7–0.8 MPa are most favorable for the formation of cartilaginous tissue. Optimal for the migration of living cells is the interstitial biological fluid pressure in the range from 20 kPa to 2 MPa (68 kPa is the most favorable value) [39]. The magnitude of distortional strain in the range from 0.05 to 1.1% (0.5% is the most favorable value) [46] contributes to formation of cartilaginous and fibrous tissues.

### 2.3. Model Verification

**Figure 4:**
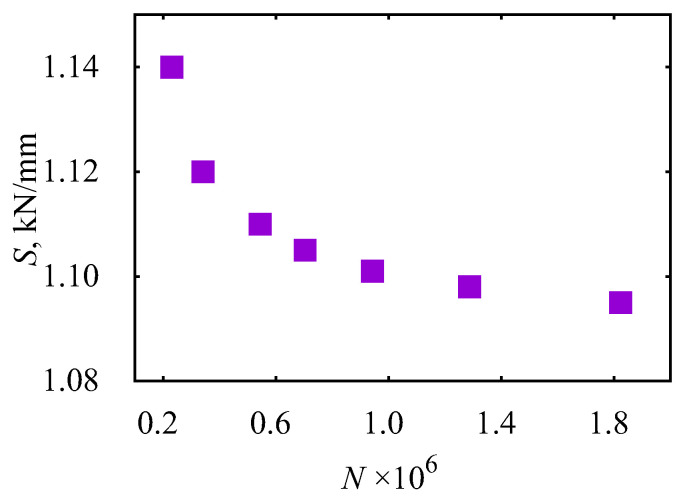
Stiffness versus number of automata in the model sample.

In this paper, by verification of the model of the mandibular segment with an implant, we mean the study of the convergence of the stiffnesses (Figure 4) of the models with different occurrences of discretization under physiological load with a maximum force of 100 N (Figure 3a).

The number of automata in the model sample ranged from 230,479 to 1,823,171 elements (with automaton diameters ranging from 400 to 200 microns correspondingly). The results on the convergence of the stiffness value of the model showed that the maximum difference in stiffness value between the minimum and maximum discretization does not exceed 4%. This difference is less than 0.5% for 942,782 and 1,823,171 automata (Figure 4), indicating a good convergence of the numerical model. However, the computation times on the computer with Threadripper 5995WX processor for these systems are 35 and 72 h, respectively. Therefore, the sample with the number of automata 942,782 (automaton diameter of 250 microns) was chosen as the optimal one for the following calculations.

### 2.4. Model Validation

**Figure 5:**
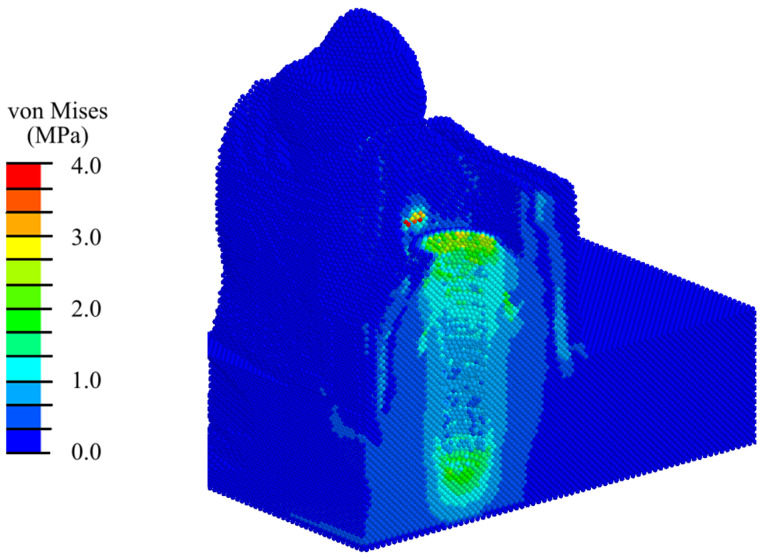
Distribution of the von Mises stress in the mandibular segment with an implant under a physiological load of 100 N.

A direct validation of the model considered herein is not possible due to the lack of an exactly similar model and corresponding experiments in the literature. However, it is worth noting that the stiffness value obtained during the verification corresponds to the literature data with a good accuracy (the authors of work [47] mentioned that the vertical settlement of natural teeth could reach 28 µm at 10 pounds load, which corresponds to a stiffness of up to 1.6 kN/mm). The validation for the main local characteristics (stresses around the implant) was carried out against a similar model with an implant found in the literature [48,49]. For this purpose, we simulated a physiological compressive load of 100 N on the implant and analyzed the distribution of the von Mises stresses in the bone around the implant (Figure 5). The comparison of the von Mises stresses in Figure 5 and the corresponding distribution in Figure 8 from [48] and Figure 6 from [49] allows us to conclude that our model is validated against data from the literature.

### 2.5. Restrictions of the Model

As the main focus of the study was on the processes occurring in the jawbone, a simplified model of the tooth was used, consisting of a homogeneous material—dentin. We only assumed the cementless placement of the implant, which is currently used in the vast majority of cases. The process of osseointegration of a thin implant prior to crown placement was considered. A case was modeled with a peri-implant gap of the order of 0.5 mm filled with fibrous-like tissue whose properties corresponded to different phases of osseointegration.

Due to the approach used, the model presented herein is not capable of predicting how long the implant osseointegration will take under a particular shock wave exposure. Such estimations could be made using mechanoregulatory model. However, as mentioned above, the coefficients for using this model in the conditions of pulse loading have not been defined so far, since there are no corresponding experimental data.

In this study, we focused on finding the intensity values of ESWT that promote osseointegration of dental implants. Instead of using mechanoregulatory model to predict changes in surrounding tissue properties, we considered three main phases of typical implant integration wherein the material around the implant has corresponding properties. Considering the final result that the optimal intensity is approximately the same in all three phases of integration, it is not so critical to know the exact duration of the process so far. However, incorporating mechanoregulatory model could prove advantageous for the implementation of the proposed approach in clinical practice.

## 3. Results

### 3.1. Modeling at the Initial Phase of Osseointegration

During the first phase of osseointegration, the peri-implant gap (the shell around the implant in our model) is filled with the primary matrix. The primary matrix (blood clot with a meshwork of fibrin fibers) was modeled as a highly permeable porous framework, with similar properties as a fibrous tissue (Table 1).

Preliminary analysis of the modeling results showed that, under therapeutic shock wave loading in all phases of osseointegration, shear strains in the peri-implant region did not exceed 0.05%. Since, according to mechanobiological principles, higher deformations are required for the formation of undesirable fibrous tissue, only two parameters will be considered in the work below: hydrostatic pressure and pressure of biological interstitial fluid (in terms of poroelasticity: pore fluid pressure).

In order to evaluate the volume ratio of the peri-implant zone where the conditions for the development of specific tissue in accordance with mechanobiological principles are met, we calculated the number of corresponding automata during shock wave passage and divided it by the total number of automata in the zone. Given the slight fluctuations in this ratio over time, we employed a 5% mean value to ensure greater precision in our calculations.

#### 3.1.1. Physiological Loading

To analyze the effectiveness of shock wave exposure in order to accelerate the implant osseointegration, the developed model of the mandible was also considered under physiological loads simulating the process of rest (100 N) and mastication (200 N).

**Figure 6:**
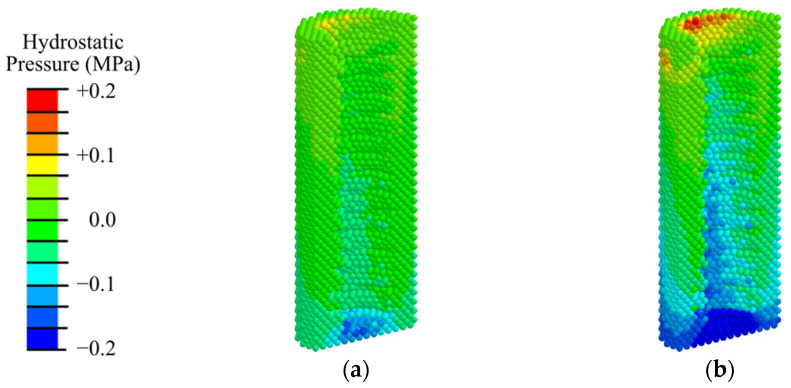
Distribution of hydrostatic pressure in the peri-implant zone in the first phase of osseointegration under physiological loads of 100 N (**a**) and 200 N (**b**).

Analysis of the hydrostatic pressure distribution (Figure 6) revealed that, under physiological loading similar to rest and mastication, conditions for bone cells differentiation are observed throughout the model specimen and the primary matrix along the interface with the implant. In this case, the maximum compressive stress is concentrated near the apex of the implant. An area of tensile stress above 0.2 MPa is formed at the surface of the periodontal zone, which is undesirable and promotes the growth of fibrous tissue.

**Figure 7:**
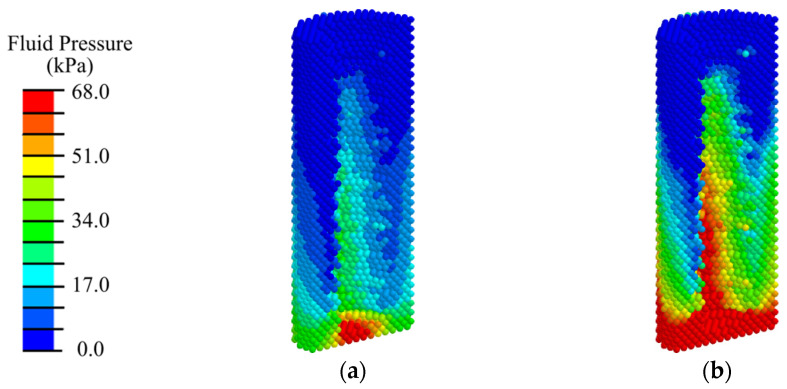
Distribution of pore fluid pressure in the peri-implant zone in the first phase of osseointegration under physiological loads of 100 N (**a**) and 200 N (**b**).

The analysis of the pore fluid pressure distribution during the modeling of the rest state (Figure 7a) showed that the required level of fluid pressure is observed in the area of the apical part of the implant in a volume of no more than 5% of the total peri-implant zone. When analyzing the results of mastication modeling (Figure 7b), it was found that the optimal level of fluid pressure is observed in the same area in 15% of the total volume of the primary matrix in the peri-implant zone. A sufficient level is observed in the lower region of the peri-implant zone.

Thus, the results of modeling the physiological loading of the mandibular segment with a premolar implant showed that such loading creates conditions for osseointegration as a gradual process. This process initiates in the distal end of the implant and gradually moves upward along its length.

#### 3.1.2. Shock Wave Exposure

**Figure 8:**
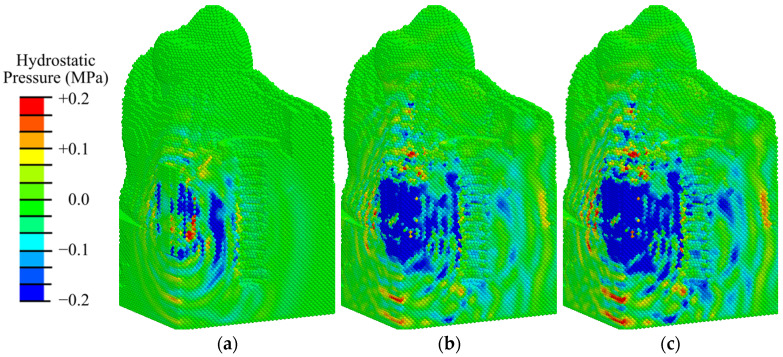
Distribution of hydrostatic pressure in the mandibular segment in the first phase of implant osseointegration under shock wave exposure with energy flux densities of (**a**) 0.02, (**b**) 0.15, and (**c**) 0.26 mJ/mm^2^.

Analysis of the results of modeling ESWT on the considered mandibular segment with an implant at the initial stage of implant integration (primary matrix with fibrin fibers) shows that the maximum compressive stresses reach 0.9 MPa at an EFD of shock wave of 0.02 mJ/mm^2^ (Figure 8a and Figure S1a); 1.2 MPa at 0.05 mJ/mm^2^ (Figure S1b); 1.3 MPa at 0.15 mJ/mm^2^ (Figure 8b and Figure S1c); 1.6 MPa at 0.26 mJ/mm^2^ (Figure 8c and Figure S1d); 1.9 MPa at 0.41 mJ/mm^2^ (Figure S1e). These are observed in the gingival tissues. The obtained values of hydrostatic pressure are significantly lower than the critical values for destruction of biological tissues.

At shock waves with an EFD less than or equal to 0.15 mJ/m^2^, regions of hydrostatic tensile stress higher than 0.2 MPa are observed locally around the implant abutment (Figure 8a,b). According to mechanobiological principles, such a stress level contributes to the differentiation of fibrous tissue cells (fibroblasts) and bone tissue cells (osteoblasts), which contributes to the formation of a fibrous matrix filled with bone tissue cells, which is the initial stage of bone tissue formation. Favorable conditions for bone tissue cell division are formed in the gingival region. Such conditions are a precursor of fibroosteointegration, which is considered a normal option in implantology. As the ESWT energy is increased, areas with tensile and compressive stresses higher than 0.2 MPa expand, leading to active differentiation of chondrocytes and fibroblasts along the implant (Figure 8c and Figure S1e). In the area of bone tissues, the maximum amplitude of compressive stresses is in the range of 0.01 to 0.2 MPa, which in turn is a favorable condition for differentiation of bone cells (osteoblasts) and favors the intramembranous type of ossification. The creation of such conditions leads to connective tissue integration, which occurs when the implant surface is surrounded by fibrous connective tissue, which is an undesirable process.

**Figure 9:**
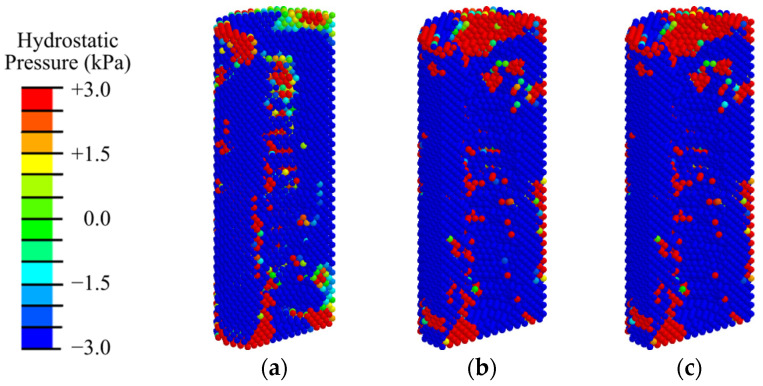
Distribution of hydrostatic pressure in the peri-implant zone of the mandibular segment in the first phase of implant osseointegration under shock wave exposure with energy flux densities of (**a**) 0.02, (**b**) 0.15, and (**c**) 0.26 mJ/mm^2^.

Nevertheless, in the peri-implant zone, even at low intensity of ESWT, the hydrostatic stresses are higher than 3 kPa, which means that chondrogenesis and osteogenesis occur there (Figure 9 and Figure S2, where the pressure scale is specially reduced to see the expansion of small pressure regions).

For the purposes of further analysis, it is convenient to divide the entire range of the studied ESWT energy flux density (0.01–0.5 mJ/mm^2^) into three parts, which hereafter will be referred to as low (0.01–0.05), medium (0.05–0.2), and high (0.2–0.5) intensities.

Thus, the analysis of the hydrostatic pressure distributions in the primary matrix showed that low- and medium-intensity shock wave exposure is favorable for direct osseointegration. High-intensity exposure can create conditions for distance osseointegration of the implant, i.e., the formation of connective tissue sections of considerable thickness between the bone and the implant.

**Figure 10:**
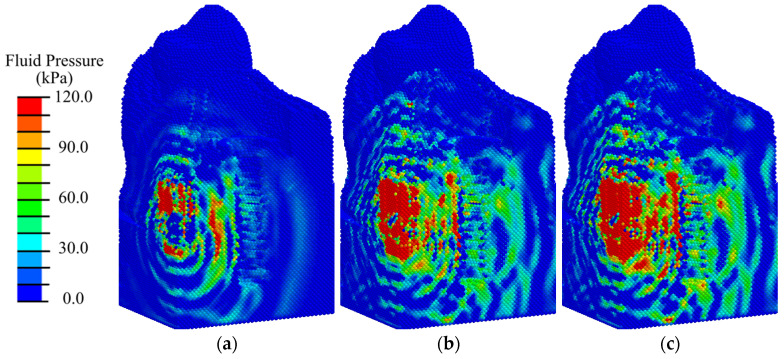
Distribution of biological fluid pressure in the mandibular segment in the first phase of implant osseointegration under shock wave exposure with energy flux densities of (**a**) 0.02, (**b**) 0.15, and (**c**) 0.26 mJ/mm^2^.

**Figure 11:**
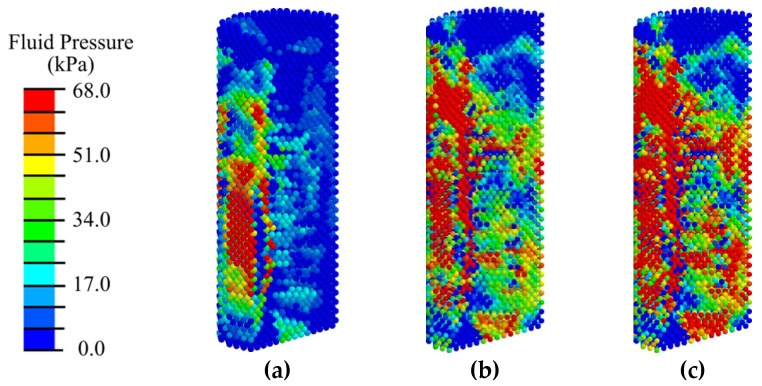
Distribution of biological fluid pressure in the peri-implant zone of the mandibular segment in the first phase of implant osseointegration under shock wave exposure with energy flux densities of (**a**) 0.02, (**b**) 0.15, and (**c**) 0.26 mJ/mm^2^.

Analysis of the distribution of the biological fluid pressure showed that with low-intensity ESWT (EFD < 0.05 mJ/mm^2^), the minimum required for cell transfer (20 kPa) from the existing bone volume is observed only in 40% of the peri-implant area, mainly in the vicinity of the load application (Figure 10, Figure 11, Figures S3, and S4). Such conditions are insufficient for the active transport of differentiable cells from the existing bone and the transition to the next stage of implant integration.

With medium- and high-intensity ESWT, optimal conditions for biological cell transfer (fluid pressure of 68 kPa) are observed in 80% of the volume of the peri-implant region consisting of blood clot. Insufficient fluid pressure is observed in 10% of the surrounding area of cortical bone. Shifting the applicator position higher does not significantly change the fluid pressure distribution pattern and does not create conditions for biological cells to transfer to the area around the abutment for their subsequent differentiation.

### 3.2. Modeling Shock Wave Exposure at the Second Phase of Osseointegration

During the second phase of osseointegration, the peri-implant gap is filled with granulation tissue. However, we also assume the formation of a thin (0.25 mm) layer of fibrous tissue directly at the interface with an implant. The properties of the granulation and fibrous tissues are shown in Table 1.

**Figure 12:**
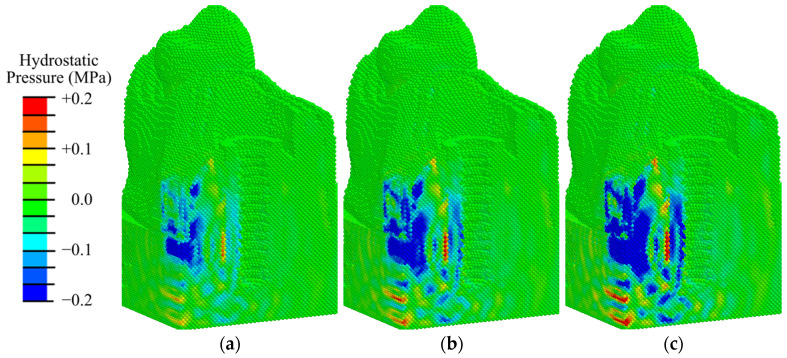
Distribution of hydrostatic pressure in the mandibular segment in the second phase of implant osseointegration under shock wave exposure with energy flux densities of (**a**) 0.02, (**b**) 0.15, and (**c**) 0.26 mJ/mm^2^.

Analysis of the hydrostatic pressure fields (Figure 12, Figures S5, and S6) shows that the values obtained in the second phase are lower than those in the first phase of osseointegration (Figure 8).

The analysis of hydrostatic pressure fields obtained for this phase according to mechanobiological principles shows that at low-intensity exposure (EFD < 0.05 mJ/mm^2^) in 75% of the volume of the peri-implant zone there are conditions for differentiation of bone tissue cells (0.003 < pressure < 0.2 MPa), which contributes to intramembranous ossification (Figure 12a). At low- and medium-intensity impacts (0.05–0.15 mJ/mm^2^), there are favorable conditions for osteoblast differentiation already in 80% of the volume of the examined area (Figure 12b and Figure S6b,c). In the area of the fibrous layer, there are no conditions for its degeneration into coarse fibrous tissue, so osteoblast differentiation also occurs in the immediate vicinity of the implant. At an ESWT EFD of 0.26 mJ/mm^2^, compressive stresses higher than 0.2 MPa are observed in the fibrous layer, which are favorable for chondrocyte formation and endochondral ossification (Figure 12c and Figure S6d). When the amplitude is increased to 0.41 mJ/mm^2^, the volume under such conditions expands (Figure S6e). In the remaining volume of granulation and fibrous tissue, conditions favorable to osteoblast differentiation are observed.

Thus, the analysis of the hydrostatic pressure distributions in the second phase of osseointegration (granulation tissue with thin layer of fibrous tissue) showed that favorable conditions for intramembranous ossification are observed at medium-intensity loading. However, at high-intensity shock wave, an area with favorable conditions for the formation of coarse connective tissue and endochondral ossification appears, which is undesirable.

**Figure 13:**
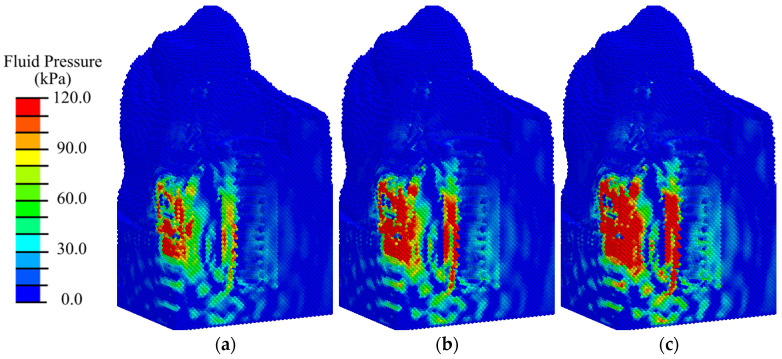
Distribution of biological fluid pressure in the mandibular segment in the second phase of implant osseointegration under shock wave exposure with energy flux densities of (**a**) 0.02, (**b**) 0.15, and (**c**) 0.26 mJ/mm^2^.

**Figure 14:**
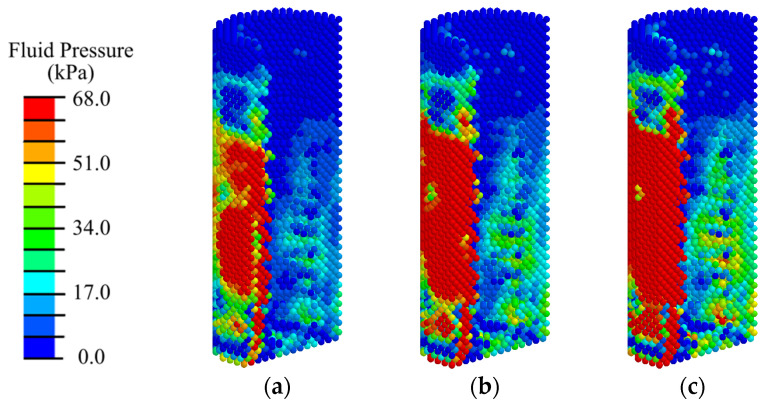
Distribution of biological fluid pressure in the peri-implant zone of the mandibular segment in the second phase of implant osseointegration under shock wave exposure with energy flux densities of (**a**) 0.02, (**b**) 0.15, and (**c**) 0.26 mJ/mm^2^.

Analysis of the distribution of the biological fluid pressure showed that, with low-intensity ESWT (EFD ≤ 0.05 mJ/mm^2^), the conditions required for cell transfer from the existing bone volume is observed only in 50% of the peri-implant area and gingiva (Figure 13 and Figure 14). With medium-intensity ESWT (EFD = 0.15 mJ/mm^2^), the conditions for biological cell transfer are observed in 80% of the volume of the peri-implant zone.

With high-intensity shock wave exposure (EFD ≥ 0.2 mJ/m^2^), the minimum level required to activate the biological cell transfer is observed in 90% of the volume of the peri-implant zone, consisting of granulation tissue and fibrous tissue (Figure 14 and Figure S8). Insufficient fluid pressure is observed in 5% of the granulation tissue volume at the height of the cortical tissue (Figure 13c and Figure S8d,e). Similar to the first phase, shifting the applicator position higher does not significantly change the fluid pressure distribution pattern and does not create conditions for biological cells to transfer to the area around the abutment for their subsequent differentiation.

### 3.3. Modeling the Final Phase of Osseointegration

As mentioned above, the third phase of osseointegration is the longest and is characterized by the restructuring of woven bone into mature bone tissue. Since mechanical stress is very important during this period, we simulated the mechanical behavior of the model under both physiological loading and shock wave exposure. It is worth noting that the elastic modulus of woven bone is lower than that of even mature cancellous bone, and the permeability is much higher than that of cortical bone, as shown in Table 1.

#### 3.3.1. Physiological Loading

**Figure 15:**
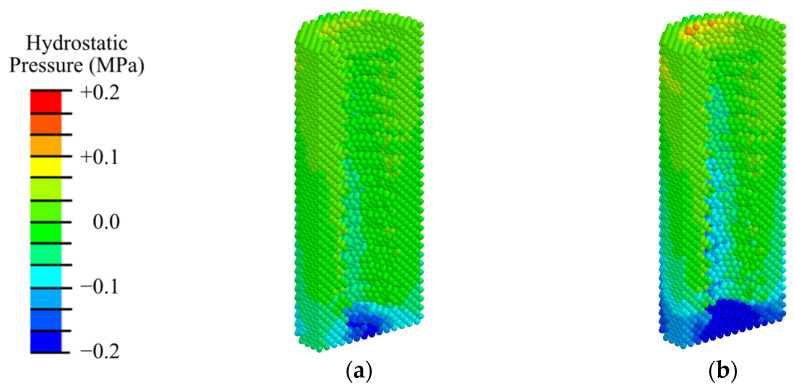
Distribution of hydrostatic pressure in the peri-implant zone during the third phase of implant osseointegration under physiological loads of 100 N (**a**) and 200 N (**b**).

Analysis of the hydrostatic pressure distribution (Figure 15) revealed that, under physiological loading similar to rest (100 N) and mastication (200 N), conditions for bone cell differentiation are observed throughout the model specimen and in the woven bone and fibrous tissue layer along the interface with the implant. Similar to the first phase (Figure 6), the maximum compressive stress is concentrated near the apex of the implant. In contrast to the first phase, the tensile stress near the abutment does not reach 0.2 MPa, indicating that during the fibrous tissue does not grow during mastication in the third phase.

**Figure 16:**
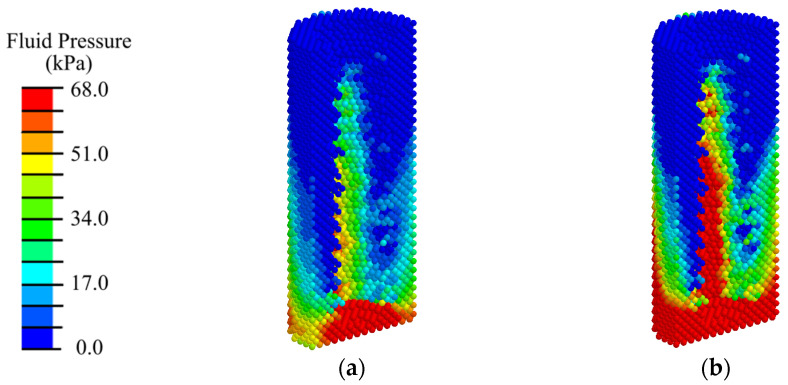
Distribution of pore fluid pressure in the peri-implant zone during the third phase of implant osseointegration under physiological loads of 100 N (**a**) and 200 N (**b**).

The pore fluid pressure in this phase (Figure 16) is approximately 5% higher than in the first phase (Figure 7). Again, in the rest state (Figure 16a), the level of fluid pressure required for cell transfer and nutrition is observed only in the distal end of the implant in a volume of about 8% of the total peri-implant zone. During mastication (Figure 16b), the optimal fluid pressure level is observed in approximately 20% of the total volume of the peri-implant zone.

Thus, the results of modeling the physiological loading of the mandibular segment with a premolar implant during the third phase of integration confirmed the results obtained for the first phase that such loading creates conditions for osseointegration as a gradual process that only slightly increases during the last phase.

#### 3.3.2. Shock Wave Exposure

**Figure 17:**
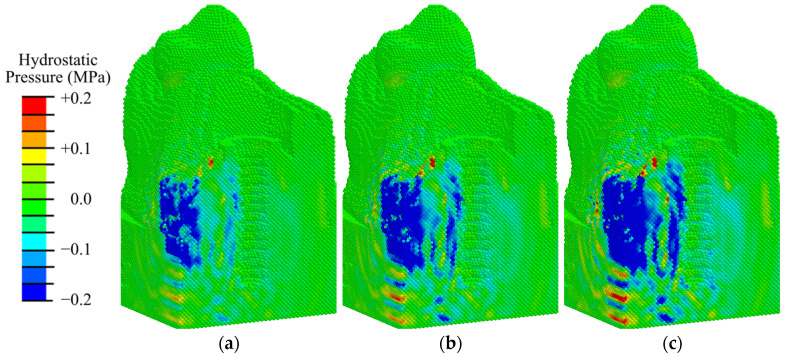
Distribution of hydrostatic pressure in the mandibular segment in the third phase of implant osseointegration under shock wave exposure with energy flux densities of (**a**) 0.02, (**b**) 0.15, and (**c**) 0.26 mJ/mm^2^.

Analysis of the hydrostatic pressure fields (Figure 17, Figure S9, and S10) shows that the level of this parameter is also higher than in the second phase of osseointegration but lower than in the first phase. The maximum compressive stresses reach 0.8 MPa at an EFD of shock wave of 0.02 mJ/mm^2^ (Figure 17a); 1.1 MPa at 0.05 mJ/mm^2^ (Figure S9b); 1.2 MPa at 0.15 mJ/mm^2^ (Figure 17b and Figure S9c); 1.6 MPa at 0.26 mJ/mm^2^ (Figure 17c and Figure S9d); 1.8 MPa at 0.41 mJ/mm^2^ (Figure S9e). These are observed in the gingival tissue. As can be seen in Figure 17 and Figure S10, hydrostatic pressure values in 98% of the volume of fibrous bone tissue surrounding the implant at the remodeling stage exceed the minimum required to create favorable conditions for bone cell differentiation (3 kPa).

At an EFD of ESWT greater than 0.1 mJ/mm^2^, extensive areas with compressive stresses greater than 0.2 MPa are observed in the woven bone area (greater than 20% of the volume of the peri-implant zone, Figure 17b,c and Figure S10c–e). Such compressive stress values are favorable for chondrocyte differentiation, which implies endochondral ossification. In the rest of the peri-implant zone, favorable conditions are observed for osteoblast differentiation, i.e., intramembranous ossification. A further increase in shock wave energy results in volume expansion with favorable conditions for cartilage cell division, which can lead to the formation of a thick layer of fibrous tissue (Figure S10c–e).

**Figure 18:**
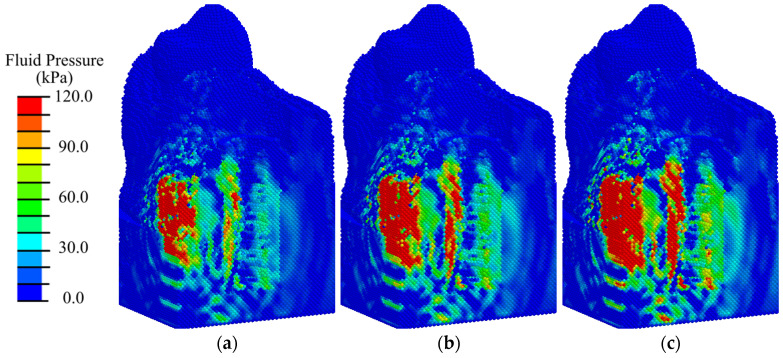
Distribution of biological fluid pressure in the mandibular segment in the third phase of implant osseointegration under shock wave exposure with energy flux densities of (**a**) 0.02, (**b**) 0.05, (**c**) 0.15 mJ/mm^2^.

An analysis of the distribution of biological fluid pressure showed that, with low-intensity ESWT (EFD ≤ 0.05 mJ/mm^2^), the condition required for cell transfer from the existing bone volume is observed in 80% of the peri-implant area in the remodeling phase (Figure 18a and Figure S12a,b). Increasing the intensity of ESWT does not expand the area with conditions for biological cell transfer, but only increases the maximum value of fluid pressure (Figure 18b,c, Figures S11c–e, and S12c–e).

## 4. Discussion of the Results

Analysis of the simulation results revealed that the conditions necessary for osseointegration are created even with a low physiological load mimicking rest in the developed mandibular model with a second premolar implant. A higher load mimicking the masticatory process is more favorable for indirect integration, with the formation of a thin layer of fibrous tissue around the implant. Under any physiological load, the conditions for bone tissue regeneration are created only around the distal end of the implant in all phases of its integration. Within the framework of the developed model, this result can be interpreted as follows. Under physiological loading, the osseointegration of the implant starts from its distal part, as this is where the best conditions exist in the initial phase. As bone tissue forms and attaches to the implant, its poroelastic properties change. However, as the calculations of the third phase showed, a change in these properties does not mean that the physiological load is able to create conditions for osteoblast differentiation higher up, towards the middle of the implant. However, the new bone will continue to grow towards the available space due to its development and the usual remodeling process. In other words, osseointegration will slowly move from the apical part of the implant along its length towards the cortical tissue. In general, the result obtained corresponds to dental practice as well as to the modeling data presented in [24], which indicates the correctness of the developed model. In a relatively small area around the implant, conditions are observed for the formation of fibrocartilaginous tissue, which can lead to distance integration.

Analysis of the data obtained for shock wave exposure on the mandibular segment model at the first phase of osseointegration of the dental implant showed that at this stage, ESWT with an energy flux density in the range of 0.05–0.15 mJ/mm^2^ is preferable. In this case, favorable conditions for direct integration are observed. At a lower intensity of shock wave exposure, conditions for osteoblast differentiation are also created, but conditions for the transfer of differentiable cells from old bone tissue are observed only in a small volume of the peri-implant zone. At higher intensities of ESWT, the formation of a connective tissue layer around the implant is possible (distance integration).

In the second phase of osseointegration, ESWT with an energy flux density in the range of 0.05 to 0.15 mJ/mm^2^ was demonstrated to facilitate the differentiation of osteoblasts in granulation tissue. A high-intensity shock wave (>0.15 mJ/mm^2^) has been observed to facilitate the process of an endochondral type of ossification (through cartilaginous tissue) on the interface of the peri-implant zone in proximity to the shock wave applicator. Concurrently, the thin fibrous layer surrounding the implant may undergo alterations, leading to increased tissue stiffness and thickness.

During the remodeling phase, low-intensity shock wave action (<0.05 mJ/mm^2^) is optimal for facilitating the active transfer of biological cells from the surrounding old bone tissue and their subsequent differentiation into osteoblasts. The application of medium- and high-intensity ESWT results in the formation of a layer of fibrocartilage tissue at the bone–peri-implant zone interface. Additionally, it contributes to an increase in the thickness and stiffness of the fibrous layer surrounding the implant.

In 2016, Cai et al. [50] experimentally demonstrated that ESWT in the intensity range of 0.05–0.19 mJ/mm^2^ facilitates the expression of genes responsible for fibroblast differentiation, which is beneficial during the initial stages of implant engraftment. Later, an increase in osteoblast proliferation was observed experimentally at EFD up to 0.18 mJ/mm^2^ [51].

A positive effect on the proliferation of osteoblasts in the bone tissue during tooth movement in rats was observed at ESWT of 0.1 mJ/mm^2^ [52]. A positive effect on the regeneration of bone tissues around orthodontically moved teeth in rabbits was observed in vivo following exposure by shock waves with an energy flux density of 0.19 mJ/mm^2^, as reported in [53].

The beneficial impact of medium-intensity ESWT with an intensity of 0.19 mJ/mm^2^ on the differentiation of fibroblasts responsible for the formation of fibrous tissue in the initial phase of implant integration was observed by Senel et al. [54]. A review of studies on the processes of osteogenesis in the bone tissues of the maxillofacial area, presented by Özkan et al. in 2019 [55], demonstrated that ESWT in the range from 0.1 to 0.25 mJ/mm^2^ exerts a beneficial effect on the differentiation and proliferation of bone cells, in addition to exhibiting antibacterial properties. A positive antimicrobial effect of focused ESWT with EFD of 0.4 mJ/mm^2^ in a rabbit model of fracture-related infection was later reported in [56]. Nevertheless, Özkan et al. concluded that the required energy flux density, number of impulses, frequency, and pressure values for shock waves to elicit optimal biological effects remain unclear. The results of previous studies have demonstrated that the effect of ESWT depends on dose and applied tissue [55].

Heimes et al. [57] observed an improvement in conditions conducive to the transfer of healthy cells in the collagen matrix formed during the initial phase of implant integration following the application of ESWT with an intensity of 0.1 mJ/mm^2^. This resulted in increased vascularization and angiogenesis. These factors are instrumental in enhancing the osseointegration of dental implants. Conversely, the findings of the recent study [58] suggest that ESWT with an EFD of 0.1 mJ/mm^2^ exerts a favorable impact on the proliferation of osteoclasts, which are responsible for bone resorption. It is worth noting that this EFD value best matches what we found.

The most recent review of results from experimental studies indicates that an increase in bone density in the dental region exposed to ESWT is observed over a wider range of ESWT intensities of 0.19–0.5 mJ/mm^2^ (similar to those used in our study) [59]. As mentioned above, there is currently a lack of experimental data on the use of ESWT for accelerating osseointegration. In this regard, it is noteworthy that in vivo experiments have demonstrated an increase in the rate of osseointegration of dental implants inserted in the femur bones of New Zealand rabbits with shock wave exposure at an energy flux density of 0.3 mJ/mm^2^ [60], which also better corresponds to our values.

## 5. Conclusions

The results of the numerical study conducted in this paper indicate that low-intensity shock wave exposure can accelerate and promote direct osseointegration through the action of growth factors. The primary advantage of ESWT in comparison to physiological loads seems to be the creation of conditions conducive to the regeneration of biological tissues of the required type along the entire surface of the implant simultaneously. This can significantly influence the rate of implant integration during the osteoinduction phase (the initial stage of osseointegration) and, most crucially, during the longest final phase of woven bone remodeling.

Despite the fact that we have managed to obtain the optimal ranges of ESWT intensity for our model, i.e., the most rational ranges at each phase of implant integration, these data are only approximate. However, the developed computer models can form the basis for calculating the parameters of ESWT for a particular patient, taking into account their physiological characteristics, as well as the characteristics of the ESWT apparatus. In future, it is also desirable to extend our model with mechanoregulatory equations, which would be able to estimate the time of osseointegration and the development of bone/fibrous tissues over time. Thus, the results of our work provide the scientific basis for further applied research to develop personalized treatment plans in dental clinics.

## Figures and Tables

**Table 1:** Elastic and poroelastic parameters of the tissues.

Type of Tissue	Density of the Matrix, *ρ*, kg/m^3^	Young’s Modulus of the Matrix, *E*, GPa	Poisson’s Ratio, ν	Bulk Modulus of the Solid, *K*_s_, GPa	Porosity, *θ*	Permeability, *k*, m^2^
Dentin	2100	18.0	0.27	50.0	0.02	2.0 × 10^−16^
Cortical	1850	14.7	0.32	17.0	0.04	1.0 × 10^−20^
Cancellous	700	3.5	0.32	15.0	0.7	3.7 × 10^−16^
PDL	800	1.0	0.30	2.3	0.8	1.0 × 10^−15^
Fibrous	800	0.6	0.29	2.3	0.8	1.0 × 10^−17^
Primary matrix	800	0.6	0.29	2.3	0.8	1.0 × 10^−17^
Granulation	800	0.3	0.29	2.3	0.8	1.0 × 10^−17^
Woven bone	800	1.2	0.30	4.6	0.8	1.0 × 10^−16^

**Table 2:** Elastic–plastic parameters of the metal implant and shock wave applicator.

Material	Density, *ρ*, kg/m^3^	Young’s Modulus, *E*, GPa	Poisson’s Ratio, ν	Yield Stress, *σ*_y_, MPa
Ti-6Al-4V	4500	107.1	0.31	800.0
Cu	8950	111.4	0.34	80.0

## Data Availability

The data that support the findings of this study are available from the corresponding author upon reasonable request. The data are not publicly available due to privacy.

## Supplementary Materials

The following supporting information can be downloaded at: https://www.mdpi.com/article/10.3390/ma17246204/s1, Figure S1: Distribution of hydrostatic pressure in the mandibular segment in the first phase of implant osseointegration under a shock wave exposure; Figure S2: Distribution of hydrostatic pressure in the peri-implant zone of the mandibular segment in the first phase of implant osseointegration under a shock wave exposure; Figure S3: Distribution of biological fluid pressure in the mandibular segment in the first phase of implant osseointegration under a shock wave exposure; Figure S4: Distribution of biological fluid pressure in the peri-implant zone of the mandibular segment in the first phase of implant osseointegration under a shock wave exposure; Figure S5: Distribution of hydrostatic pressure in the mandibular segment in the second phase of implant osseointegration under a shock wave exposure; Figure S6: Distribution of hydrostatic pressure in the peri-implant zone of the mandibular segment in the second phase of implant osseointegration under a shock wave exposure; Figure S7: Distribution of biological fluid pressure in the mandibular segment in the second phase of implant osseointegration under a shock wave exposure; Figure S8: Distribution of biological fluid pressure in the peri-implant zone of the mandibular segment in the second phase of implant osseointegration under a shock wave exposure; Figure S9: Distribution of hydrostatic pressure in the mandibular segment in the third phase of implant osseointegration under a shock wave exposure; Figure S10: Distribution of hydrostatic pressure in the peri-implant zone of the mandibular segment in the third phase of implant osseointegration under a shock wave exposure; Figure S11: Distribution of biological fluid pressure in the mandibular segment in the third phase of implant osseointegration under a shock wave exposure; Figure S12: Distribution of biological fluid pressure in the peri-implant zone of the mandibular segment in the third phase of implant osseointegration under a shock wave exposure.

## Nomenclature

ESWT	extracorporeal shock wave therapy
EFD	energy flux density
MCA	movable cellular automaton
PDL	periodontal ligament
MPa	megapascals (10^6^ Pa) as a unit of pressure
mJ	millijoules (10^−6^ J) as a unit of energy
