# Supplementary material for: Biomechanics of Osseointegration of a Dental Implant in the Mandible Under Shock Wave Therapy: In Silico Study
Source: Materials (Basel). 2024 Dec 19;17(24):6204. doi: 10.3390/ma17246204 (PMC11677247; doi:10.3390/ma17246204)
Supplement: Supplementary file 1 [file materials-17-06204-s001.zip › materials-3358440-supplementary.pdf]

materials

MDPI

Supplementary Materials to article

Biomechanics of Osseointegration of a Dental Implant in the Mandible under Shock Wave Therapy: In Silico Study

Alexey Smolin [1,*], Galina Eremina [1], Irina Martyshina [1] and Jing Xie [2]

[1] Institute of Strength Physics and Materials Science, Siberian Branch of the Russian Academy of Sciences, Pr. Akademicheskii, 2/4, 634055 Tomsk, Russia; asmolin@ispms.ru; anikeeva@ispms.ru; mira@ispms.ru
[2] State Key Laboratory of Explosion Science and Technology, Beijing Institute of Technology, Beijing 100081, China; jxie@bit.edu.cn
* Correspondence: asmolin@ispms.ru

1. Movable cellular automaton method

In this method, the material is considered as an ensemble of discrete elements of the same finite size (movable cellular automata) that interact with each other according to certain rules, which due to many-body interaction forces describe the deformation behavior of the material as an isotropic elastoplastic body. The motion of the movable cellular automata is governed by the Newton-Euler equations for their translation and rotation:

$$\begin{cases} m_i \ddot{\mathbf{R}}_i = \sum_{j=1}^{N_i} \mathbf{F}_{ij}^{\text{pair}} + \mathbf{F}_i^{\Omega}, \\ \hat{J}_i \dot{\boldsymbol{\omega}}_i = \sum_{j=1}^{N_i} \mathbf{M}_{ij}, \end{cases} \tag{S1}$$

where $\mathbf{R}_i$, $\boldsymbol{\omega}_i$, m_i, $\hat{J}_i$ are the location vector, rotation velocity, mass and moment of inertia of i-th element, $\mathbf{F}_{ij}^{\text{pair}}$ is the pair force of interaction of the i-th and j-th elements, $\mathbf{F}_i^{\Omega}$ is the force acting on i-th element due to interaction of all its neighbors with the other elements (so called volume-dependent force). Herein, the upper dot denotes a time derivative. In the second line of equations (S1) $\mathbf{M}_{ij} = q_{ij}(\mathbf{n}_{ij} \times \mathbf{F}_{ij}^{\text{pair}}) + \mathbf{K}_{ij}$ is the total torque of pair i–j, where q_{ij} is the distance between the center of i-th element and its contact point with j-th neighbor, $\mathbf{n}_{ij} = (\mathbf{R}_j - \mathbf{R}_i)/r_{ij}$ is the unit vector directed from the center of i-th element to the j-th one and r_{ij} is the distance between centers of these elements, $\mathbf{K}_{ij}$ is the torque due to only relative rotation of the pair elements.

If the material is isotropic, the volume-dependent force can be written as follows:

$$\mathbf{F}_i^{\Omega} = -A \sum_{j=1}^{N_i} P_j S_{ij} \mathbf{n}_{ij}, \tag{S2}$$

where P_j is the hydrostatic pressure in the bulk of element j, S_{ij} is the interaction area of elements i and j, and A is the material parameter, which is determined by the ratio of the elastic moduli. Taking this into account it is possible to rewrite the total force acting on automaton i as a decomposition of normal and tangential components ($\mathbf{F}_{ij}^{\text{n}}$ and $\mathbf{F}_{ij}^{\tau}$):

$$\mathbf{F}_i = \sum_{j=1}^{N_i} \left(\mathbf{F}_{ij}^{\text{pair}} - AP_i S_{ij} \mathbf{n}_{ij} \right) = \sum_{j=1}^{N_i} \left[\left(F_{ij}^{\text{pair,n}} \left(h_{ij} \right) - AP_j S_{ij} \right) \mathbf{n}_{ij} + F_{ij}^{\text{pair},\tau} \left(\mathbf{l}_{ij}^{\text{shear}} \right) \mathbf{t}_{ij} \right] = \sum_{j=1}^{N_i} \left(\mathbf{F}_{ij}^{\text{n}} + \mathbf{F}_{ij}^{\tau} \right), \tag{S3}$$

where $F_{ij}^{\text{pair,n}}$ and $F_{ij}^{\text{pair,}\tau}$ are the corresponding components of the pair interaction force depending on the central r_{ij} and tangential $\mathbf{l}_{ij}^{\text{shear}}$ relative displacements, respectively. Using the homogenization procedure described in [33] it is possible to determine the average stress tensor in the bulk of element i as follows:

$$\bar{\sigma}_{\alpha\beta}^{i} = \frac{1}{V_i}\sum_{j=1}^{N_i} q_{ij} n_{ij,\alpha} F_{ij,\beta} \,, \tag{S4}$$

where α and β are used to denote the axes X, Y, Z of the global coordinate system, V_i is the element volume, $n_{ij,\alpha}$ is the α-component of the vector $\mathbf{n}_{ij}$, $F_{ij,\beta}$ is the β-component of the force acting between elements i and j. Having components of the stress tensor allows easy computing all its invariants including von Mises stress.

Elements of the pair may represent the parts of different solids (in this case it is a contacted pair) or of the same solid (i.e. a bonded pair, and interaction of these elements is not a real contact). The size of an element is characterized by one parameter d_i, which is an approximation because geometrically the element is determined by its areas of interaction with neighbors.

To characterize the deformation of element i due to its normal interaction with element j we use the following formula for normal strain

$$\xi_{ij} = \frac{q_{ij} - d_i/2}{d_i/2} \,. \tag{S5}$$

Each element of the pair may be made of different materials. Hence the increment of the relative displacements r_{ij} of the pair leads to different strain increments from i-th and j-th elements:

$$\Delta r_{ij} = \Delta q_{ij} + \Delta q_{ji} = \Delta\xi_{ij}\, d_i/2 + \Delta\xi_{ji}\, d_j/2 \,, \tag{S6}$$

where symbol Δ denotes an increment per time step Δt. The same is valid for the tangential displacement $\mathbf{l}_{ij}^{\text{shear}}$ and shear strain γ_{ij}. To define the strain distribution in the pair we need the rule for computing interaction force (constitutive equation). In MCA it is the same as Hooke's law for corresponding components of stress tensor:

$$\begin{cases} \Delta F_{ij}^{\text{pair,n}} = 2G(\Delta\xi_{ij}) - \left(1 - 2G/K\right)\Delta P_i \\ \Delta F_{ij}^{\text{pair,}\tau}{}_{ij} = 2G_i \Delta\gamma_{ij} \end{cases} \,, \tag{S7}$$

where K and G are the bulk and shear moduli of the material of i-th element, P_i is computed via the stress tensor components defined by equation (S4) at the previous time step and may be corrected by additional iteration.

Due to the necessity of Newton's third law, the increments of the reaction forces of the elements i and j are calculated based on the solving of the following system of equations:

$$\begin{cases} \Delta F_{ij}^{\text{pair},n} = \Delta F_{ji}^{\text{pair},n} \\ \Delta\xi_{ij}\, d_i/2 + \Delta\xi_{ji}\, d_j/2 = \Delta r_{ij} \\ \Delta F_{ij}^{\text{pair},\tau} = \Delta F_{ji}^{\text{pair},\tau} \\ \Delta\gamma_{ij}\, d_i/2 + \Delta\gamma_{ji}\, d_j/2 = \Delta l_{ij}^{sh} \end{cases} \,, \tag{S8}$$

where Δr_{ij} is the change in the distance between the centers of the elements i and j per time step Δt, Δl_{ij}^{sh} is the value of the relative shear displacement of these interacting elements. This means that discrete elements made of different materials deform differently according to their elastic properties and stress state. This ensures correct modeling

of the contact interaction of different solids as well as the interaction at the interface between the inclusion and the matrix in heterogeneous materials (composites).

To integrate equations (S1), an explicit velocity Verlet scheme is used. To make the scheme stable, the value of time step is limited by the time of sound propagation along the element.

To describe the mechanical behavior of the fluid-saturated material in the MCA method, the following effective (implicit) characteristics of the automata are introduced: the volume fraction of interstitial fluid (its density ρ), porosity ϕ, permeability k, and the ratio $a = 1 - K/K_s$ of the macroscopic value of bulk modulus K to the bulk modulus of the skeleton (solid part of the material) K_s [33]. The mechanical influence of the interstitial fluid on the stresses and strains in the solid skeleton of an element is described on the basis of the linear Biot's model of poroelasticity [33], which assumes that the mechanical response of a "dry" element is linearly elastic, and the mechanical effect of the pore fluid on the element stress can be described in terms of the local pore pressure P^{pore} (fluid pore pressure in the volume of the element), which affects only the diagonal components of the stress tensor. It means that only the relations for the central interaction in (S7) should be modified:

$$\Delta F_{ij}^{\mathrm{pair},n} = 2G_i\left(\Delta\varepsilon_{ij} - \frac{a_i \Delta P_i^{\mathrm{pore}}}{K_i}\right) - \left(1 - \frac{2G_i}{K_i}\right)\Delta P_i \,. \quad \text{(S9)}$$

The fluid pore pressure in the element is calculated based on the relationships of Biot's poroelasticity model with the use of the current density of pore fluid. Linearly compressible fluid is described by the following equation of state

$$\rho\left(P^{\mathrm{pore}}\right) = \rho_0\left(1 + \left(P^{\mathrm{pore}} - P_0\right)/K_{\mathrm{fl}}\right), \quad \text{(S9)}$$

where ρ and P^{pore} are the current pore fluid density and pressure; ρ_0 and P_0 are the equilibrium values of these parameters under standard conditions; K_{fl} is the fluid bulk modulus.

The total pore space of all elements is assumed to be interconnected, which provides the possibility of redistribution (filtration) of interstitial fluid between the interacting elements. The "driving force" of filtration is a pore pressure gradient. Neglecting gravitational effects the equation of interstitial fluid filtration in the pore space can be written as follows:

$$\phi\frac{\partial\rho}{\partial t} = K_{\mathrm{fl}}\nabla\left[\frac{k}{\eta}\nabla\rho\right], \quad \text{(S10)}$$

where η is the fluid viscosity and k is the permeability of the solid skeleton, which can be calculated from the current porosity ϕ as:

$$k = \phi d_{\mathrm{ch}}^2, \quad \text{(S11)}$$

where d_{ch} is the diameter of the filtration channel.

Equations (S9)–(S11) are solved numerically using the first-order Euler scheme for integration in time on a mesh made of the centers of the interacting elements (by analogy to finite volume method on an ensemble of discrete elements). According to the used approximations, there is no fluid transfer between elements if $\rho \leq \rho_0$.

2. Modeling results

2.1. Initial phase of osseointegration

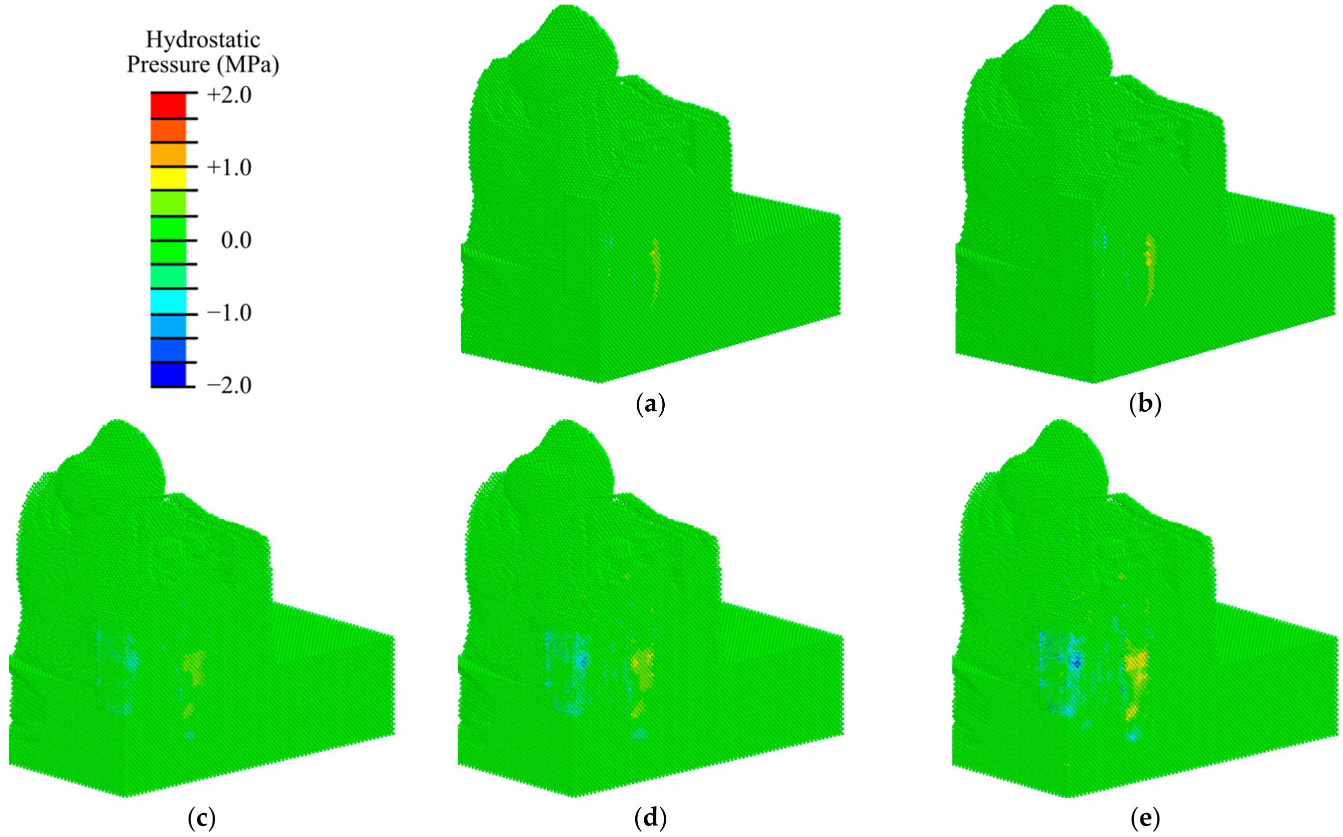

Figure S1. Distribution of hydrostatic pressure in the mandibular segment in the first phase of implant osseointegration under a shock wave exposure with energy flux densities of (**a**) 0.02, (**b**) 0.05, (**c**) 0.15, (**d**) 0.26, and (**e**) 0.41 mJ/mm^2.

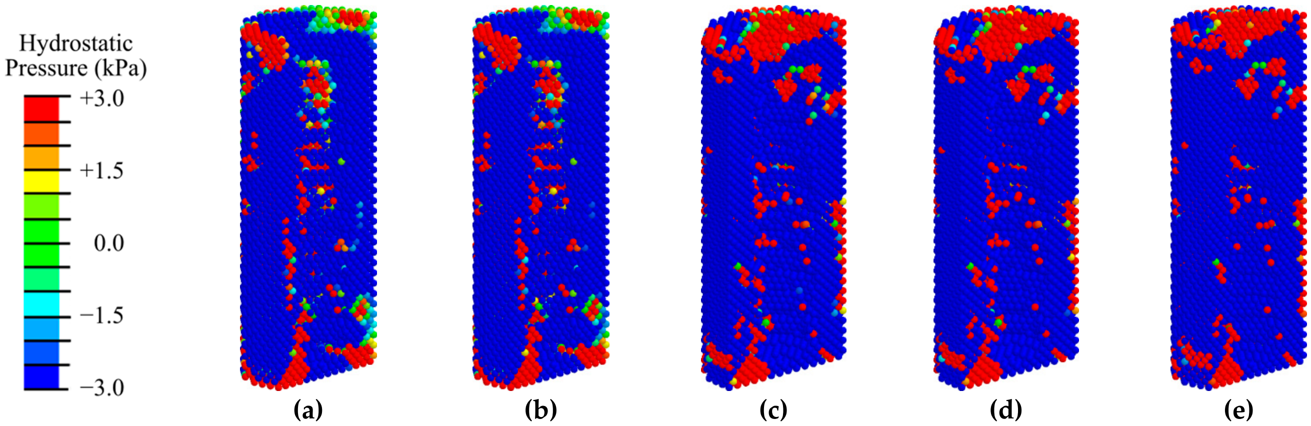

Figure S2. Distribution of hydrostatic pressure in the peri-implant zone of the mandibular segment in the first phase of implant osseointegration under a shock wave exposure with energy flux densities of (**a**) 0.02, (**b**) 0.05, (**c**) 0.15, (**d**) 0.26, and (**e**) 0.41 mJ/mm^2.

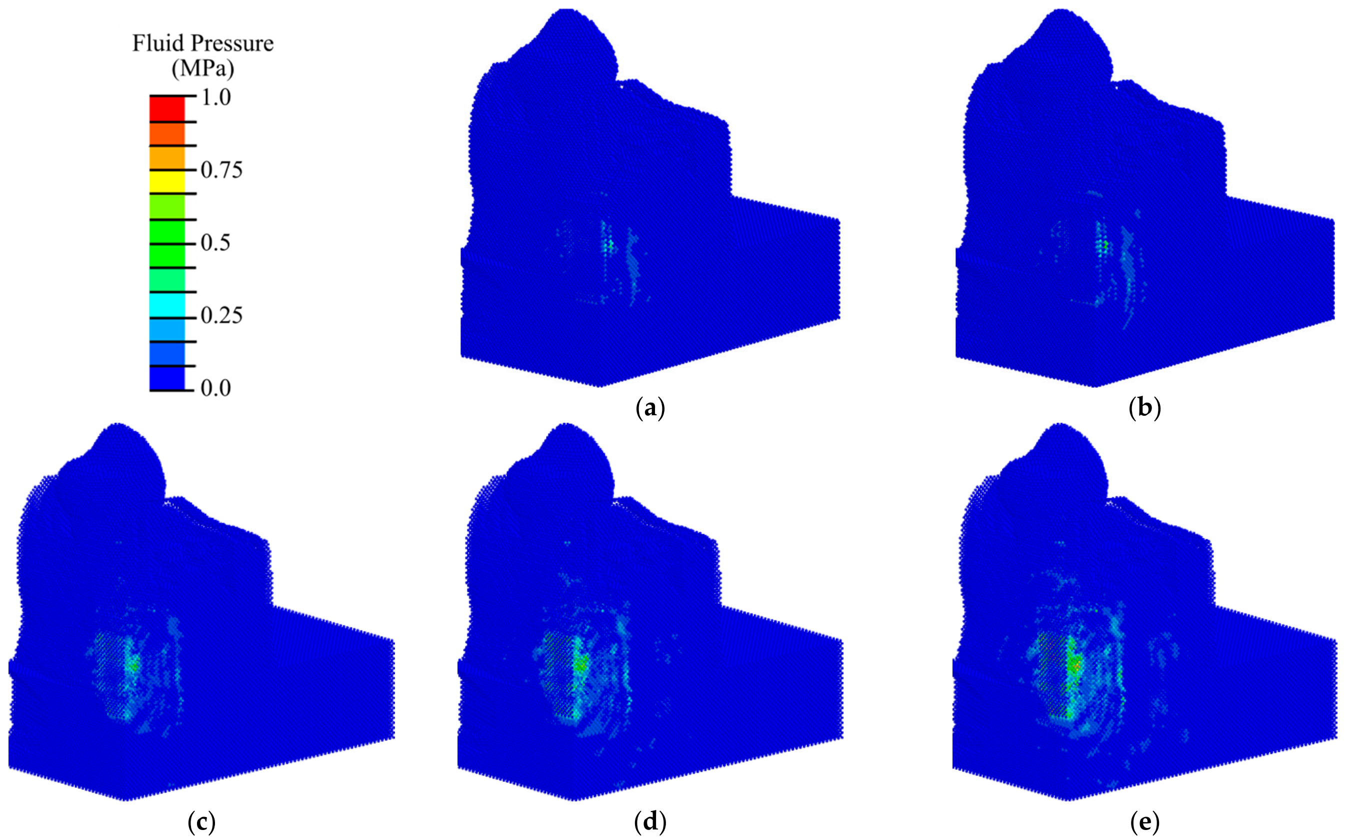

Figure S3. Distribution of biological fluid pressure in the mandibular segment in the first phase of implant osseointegration under a shock wave exposure with energy flux densities of (**a**) 0.02, (**b**) 0.05, (**c**) 0.15, (**d**) 0.26, and (**e**) 0.41 mJ/mm^2.

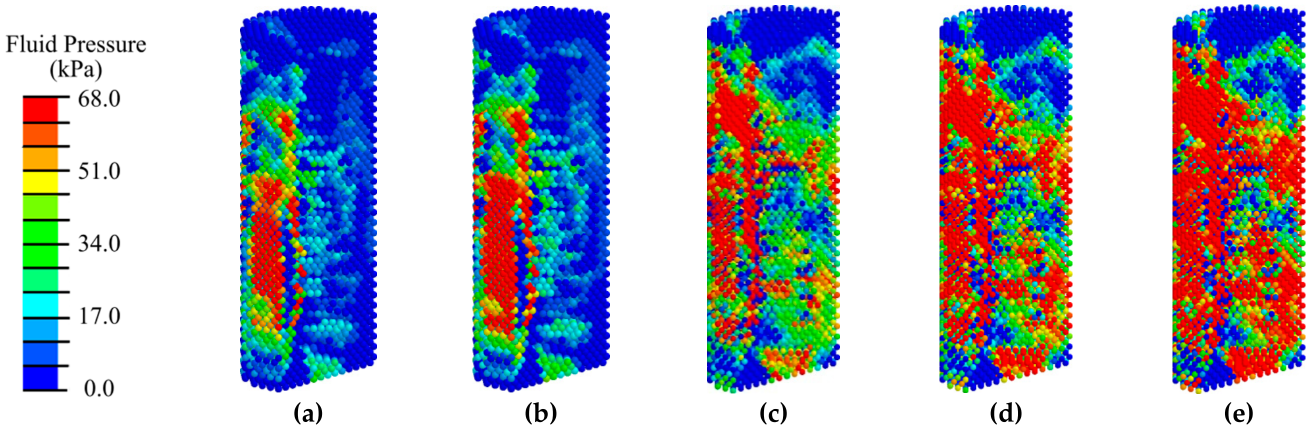

Figure S4. Distribution of biological fluid pressure in the peri-implant zone of the mandibular segment in the first phase of implant osseointegration under a shock wave exposure with energy flux densities of (**a**) 0.02, (**b**) 0.05, (**c**) 0.15, (**d**) 0.26, and (**e**) 0.41 mJ/mm^2.

2.2. Second phase of osseointegration

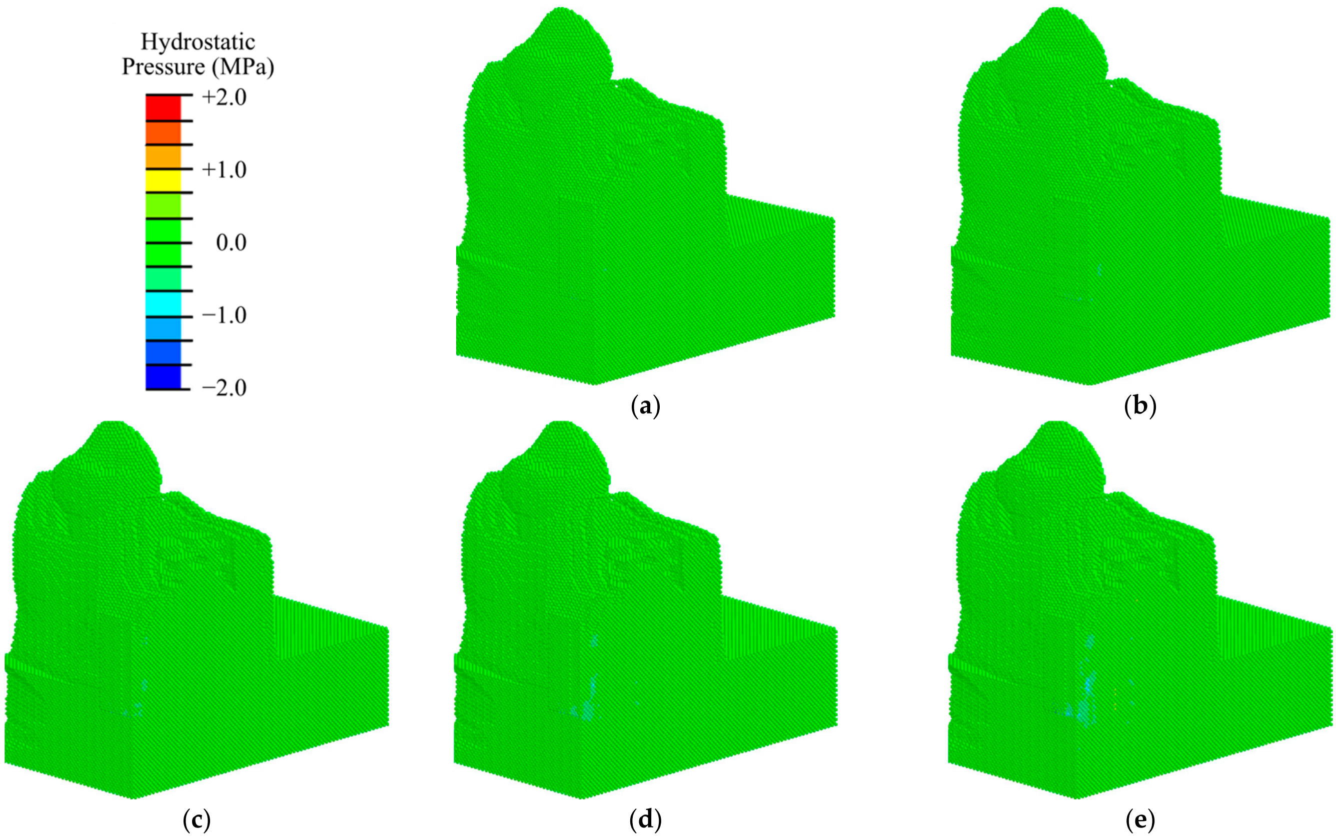

Figure S5. Distribution of hydrostatic pressure in the mandibular segment in the second phase of implant osseointegration under a shock wave exposure with energy flux densities of (**a**) 0.02, (**b**) 0.05, (**c**) 0.15, (**d**) 0.26, and (**e**) 0.41 mJ/mm^2.

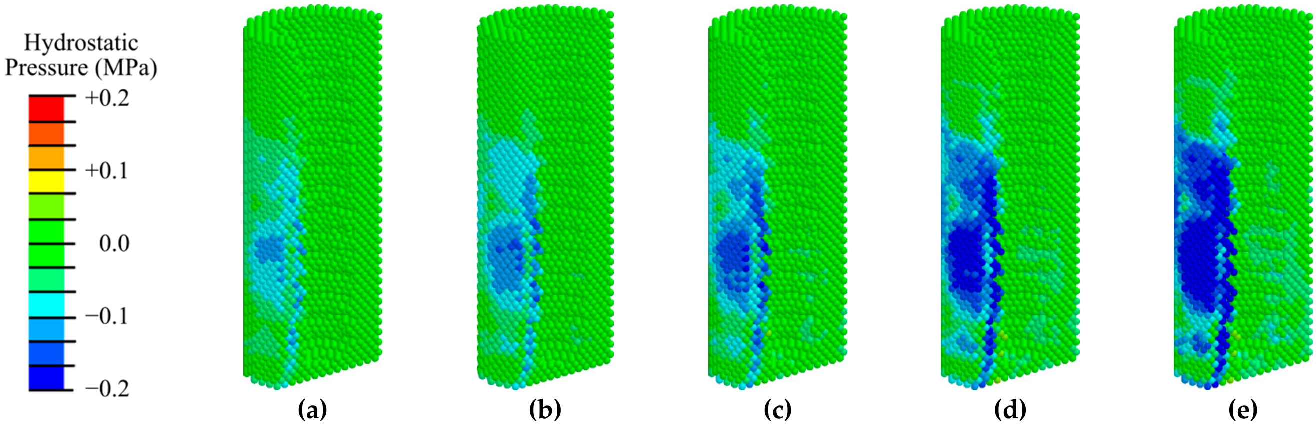

Figure S6. Distribution of hydrostatic pressure in the peri-implant zone of the mandibular segment in the second phase of implant osseointegration under a shock wave exposure with energy flux densities of (**a**) 0.02, (**b**) 0.05, (**c**) 0.15, (**d**) 0.26, and (**e**) 0.41 mJ/mm^2.

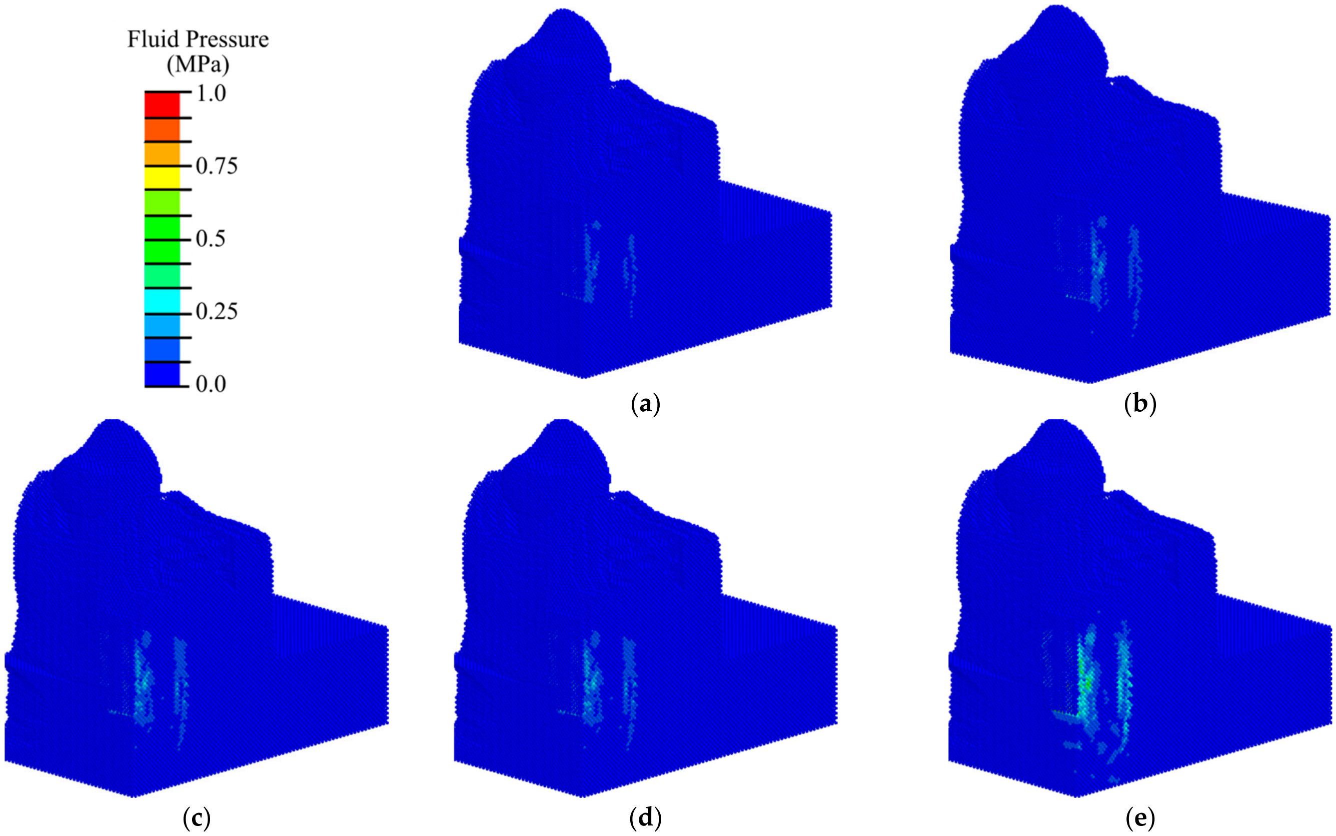

Figure S7. Distribution of biological fluid pressure in the mandibular segment in the second phase of implant osseointegration under a shock wave exposure with energy flux densities of (**a**) 0.02, (**b**) 0.05, (**c**) 0.15, (**d**) 0.26, and (**e**) 0.41 mJ/mm^2.

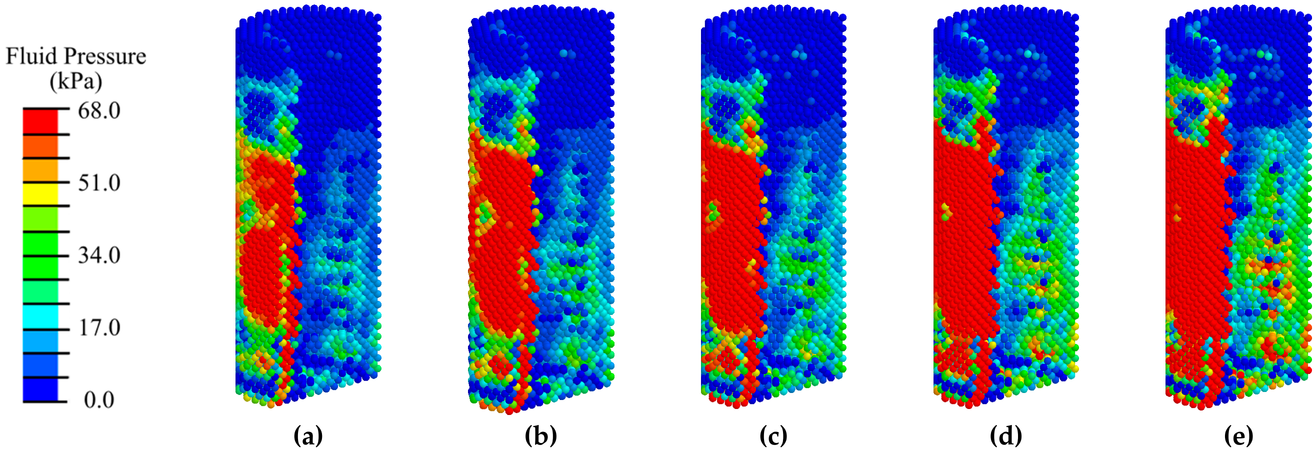

Figure S8. Distribution of biological fluid pressure in the peri-implant zone of the mandibular segment in the second phase of implant osseointegration under a shock wave exposure with energy flux densities of (**a**) 0.02, (**b**) 0.05, (**c**) 0.15, (**d**) 0.26, and (**e**) 0.41 mJ/mm^2.

2.3. Final phase of osseointegration

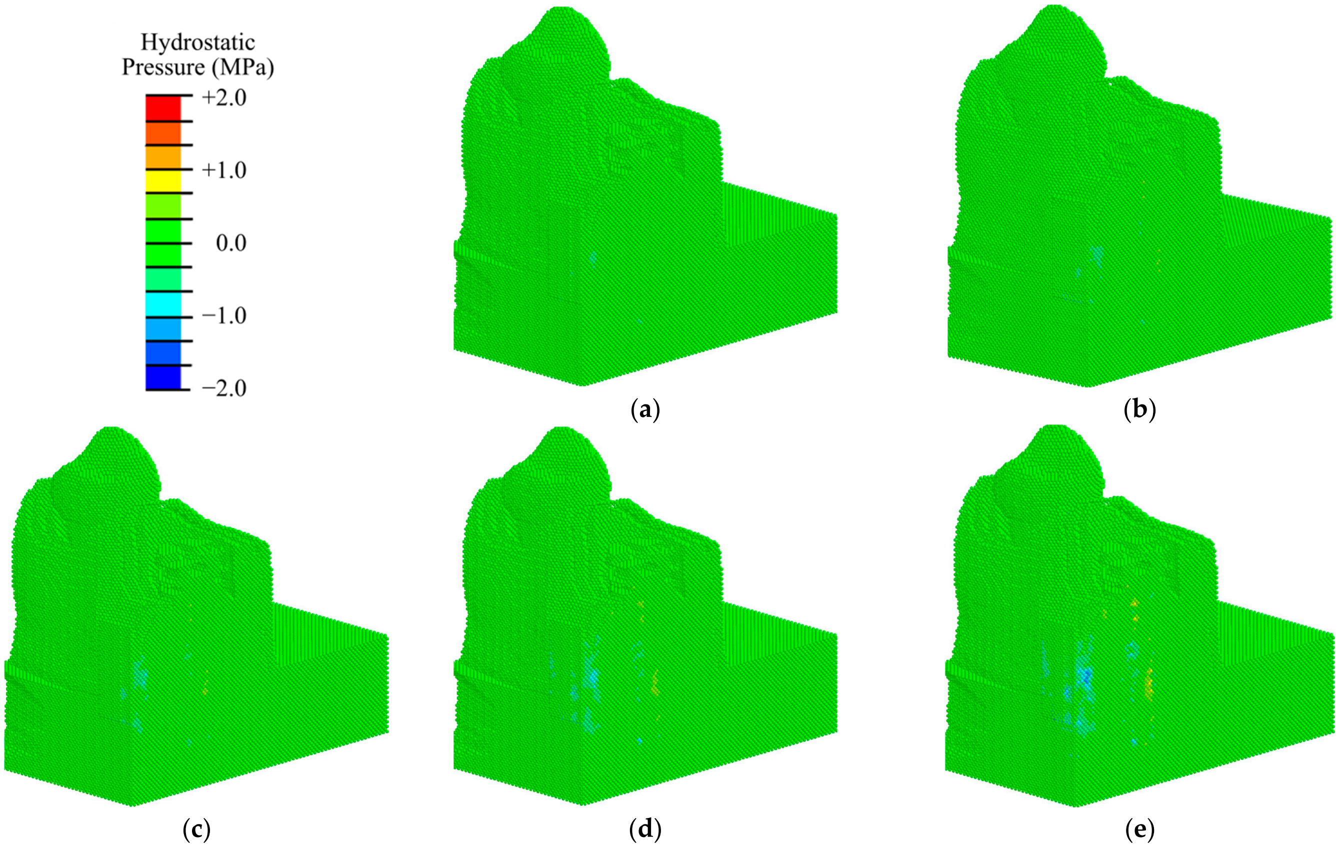

Figure S9. Distribution of hydrostatic pressure in the mandibular segment in the third phase of implant osseointegration under a shock wave exposure with energy flux densities of (**a**) 0.02, (**b**) 0.05, (**c**) 0.15, (**d**) 0.26, and (**e**) 0.41 mJ/mm^2.

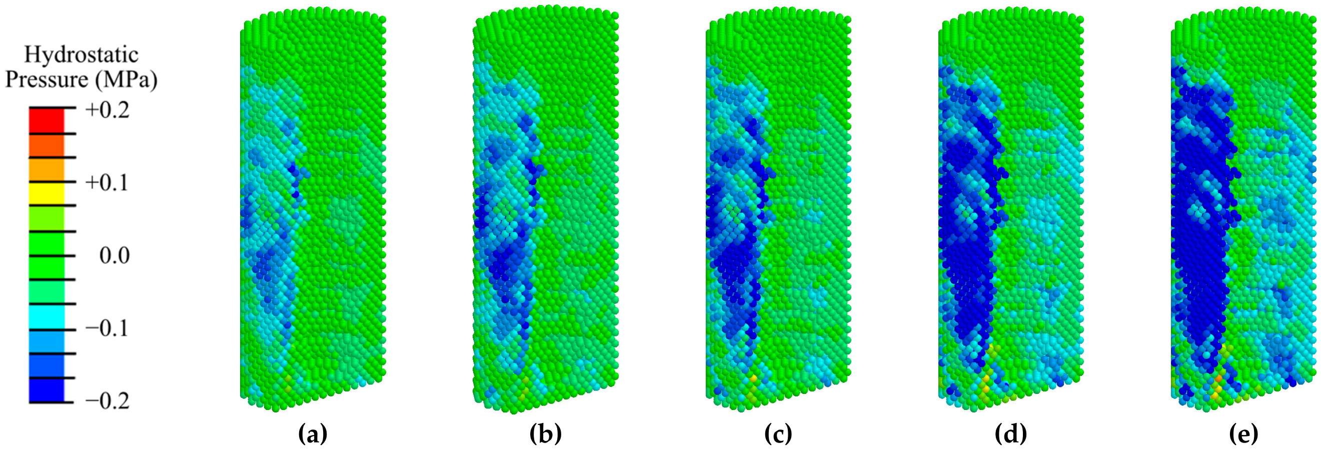

Figure S10. Distribution of hydrostatic pressure in the peri-implant zone of the mandibular segment in the third phase of implant osseointegration under a shock wave exposure with energy flux densities of (**a**) 0.02, (**b**) 0.05, (**c**) 0.15, (**d**) 0.26, and (**e**) 0.41 mJ/mm^2.

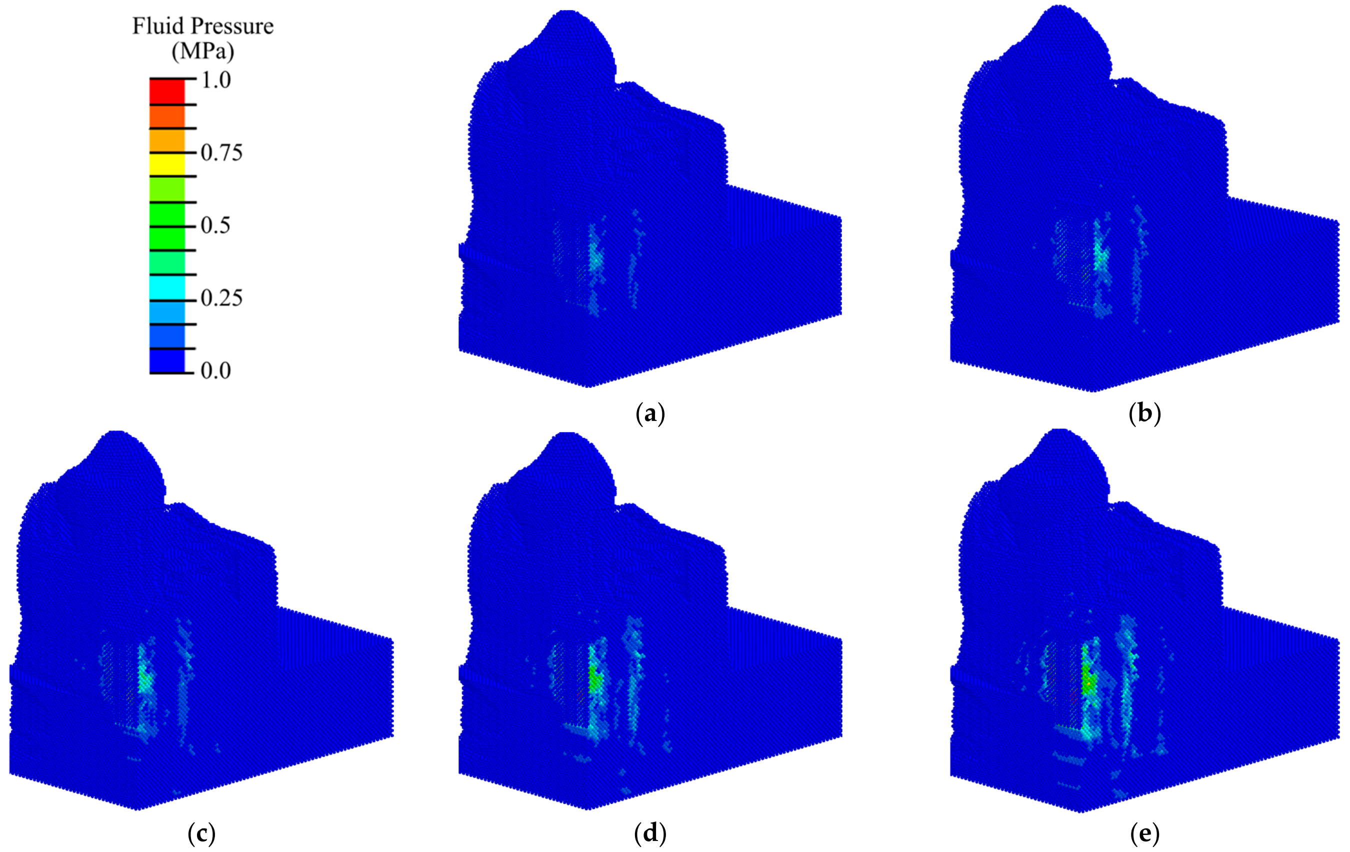

Figure S11. Distribution of biological fluid pressure in the mandibular segment in the third phase of implant osseointegration under a shock wave exposure with energy flux densities of (**a**) 0.02, (**b**) 0.05, (**c**) 0.15, (**d**) 0.26, and (**e**) 0.41 mJ/mm^2.

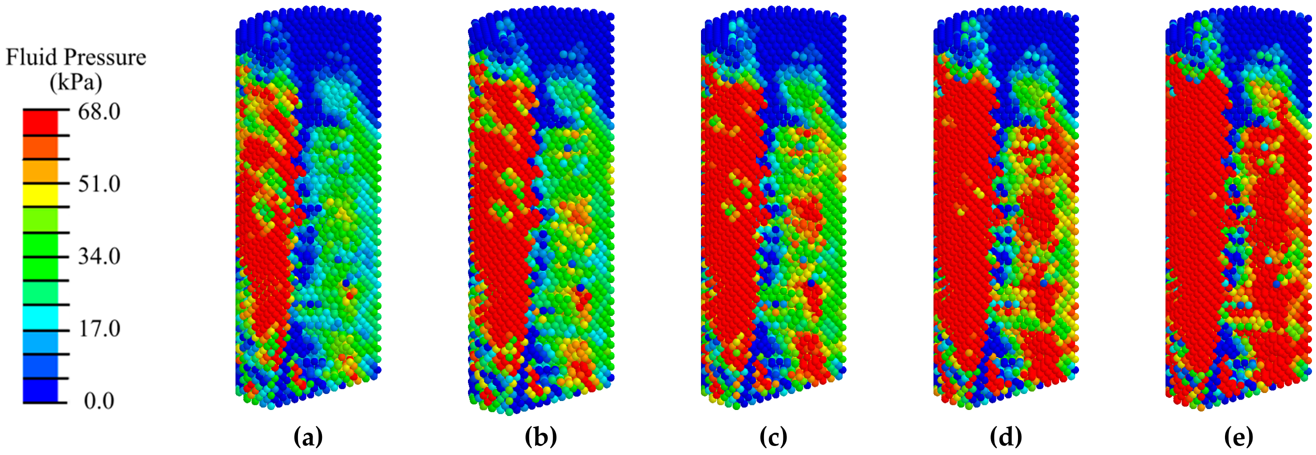

Figure S12. Distribution of biological fluid pressure in the peri-implant zone of the mandibular segment in the third phase of implant osseointegration under a shock wave exposure with energy flux densities of (**a**) 0.02, (**b**) 0.05, (**c**) 0.15, (**d**) 0.26, and (**e**) 0.41 mJ/mm^2.
